# Quantitative Supramolecular Heterodimerization for Efficient Energy Transfer

**DOI:** 10.1002/anie.202006530

**Published:** 2020-07-13

**Authors:** Guanglu Wu, Zehuan Huang, Oren A. Scherman

**Affiliations:** ^1^ Melville Laboratory for Polymer Synthesis Department of Chemistry University of Cambridge Lensfield Road Cambridge CB2 1EW UK

**Keywords:** chromophores, dimers, energy transfer, noncovalent interactions, supramolecular chemistry

## Abstract

The challenge of quantitatively forming self‐assembled heterodimers without other equilibrium by‐products is overcome through self‐sorting favored by the introduction of designed shape‐complementary moieties. Such a supramolecular strategy based on cucurbit[8]uril‐directed dimerization is further applied to generate hetero‐chromophore dimers quantitatively, leading to efficient energy transfer (>85 %) upon photoexcitation.

## Graphical abstract

**Quantitative generation of hetero‐chromophore dimers** is realized through cucurbit[8]uril‐directed self‐sorting, favored by the introduction of designed, shape‐complementary moieties. This process results in discrete heterodimeric stacking through supramolecular confinement, enabling efficient interchromophoric energy transfer upon photoexcitation.

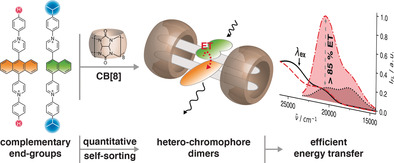

Molecular design for the precise arrangement of light‐absorbing chromophores is critical to realize the next generation of synthetic assemblies for (photo)energy applications. In the light‐harvesting systems of photosynthesis, chromophores like chlorophylls and carotenoids, are immobilized inside rigid protein scaffolds with specific alignments so as to competently capture photons and transmit the resultant excitation energy to the reaction centre.^1^, ^2^ Thus it can be seen that an efficient energy transfer requires a) an appropriate arrangement of chromophores with specifc orientation, stacking, and spacing to ensure an effective interchromophoric interaction;^3^ and b) a rigid scaffold to accommodate them so as to sustain the effective arrangement and interaction for a sufficiently long period of time.

Synthetic rigid scaffolds capable of facilitating interchromophoric coupling can be realized by connecting finite number of chromophores with appropriate spacer moieties either through covalent linkages^4^, ^5^, ^6^ or interlocked mechanical bonding.^7^, ^8^, ^9^ These approaches often result in discrete entities such as foldamers^4^, ^10^ or cyclophanes^11^, ^12^, ^13^ in solution, within which the relative arrangement of chromophores can be adjusted by the spacer,^4^, ^14^, ^15^ the solvent,^16^, ^17^ or external stimuli.^7^, ^18^ Although chromophore assemblies can also be fabricated through noncovalent interactions, they usually lead to uncontrolled aggregation in solution.^19^, ^20^ Macrocyclic hosts such as cucurbit[*n*]uril (CB[*n*], *n=*6–8, 10)^21^, ^22^, ^23^ are utilized to avoid arbitrary aggregates by encapsulating chromophores inside the cavity.^24^, ^25^, ^26^, ^27^, ^28^, ^29^, ^30^ In particular, the cavity of CB[8] is able to accommodate chromophore dimers (typically referred to as homo‐ and heteroternary complexes),^31^, ^32^ although the resultant complexes are often too dynamic to sustain long‐lived interchromophoric coupling.

Recently, we demonstrated a strategy employing noncovalent interactions to stack two chromophores, based on bis(*N*‐arylpyridinium) (BAP) derivatives, through multiple CB[8] clamping,^33^, ^34^, ^35^, ^36^ which has been applied to fabricate supramolecular complexes with emerging features such as red‐shifted absorption,^33^, ^35^ enhanced emission,^35^, ^37^, ^38^, ^39^ directional self‐sorting,^40^, ^41^ and a negative p*K*_a_ shift.^42^ More importantly, the resultant chromophore dimers typically exist as single entities for more than 30 ms. The pseudo‐static nature of these dimers in aqueous solution provides an excellent scaffold to facilitate long‐lived interchromophoric coupling.^36^ This supramolecular approach is particularly convenient to readily produce heterodimers through simple mixing unlike covalent linkages^17^ or mechanical interlocking^8^, ^9^ currently required to prepare similar heterodimers through rigorous synthesis and purification. The critical challenge for this noncovalent strategy, however, is to generate a heterodimer in a “*quantitative*” manner,^43^, ^44^ that is, without homodimers as equilibrium by‐products. In particular, amongst the reported cucurbituril‐based self‐sorting systems, the majority of them have focused on controlling orthogonal binding modes or sequence specificity.^45^, ^46^, ^47^, ^48^, ^49^, ^50^ However, few have attempted to harvest heterodimers in equilibrated systems^41^, ^51^ and none have succeeded in doing this quantitatively.

Simply mixing two homodimers (AA and BB) readily generates a heterodimer (AB), but not quantitatively, resulting in equilibrium products with a coexistence of all three species (AA, BB, and AB; Figure 1 a).^41^, ^52^, ^53^, ^54^ Two BAP derivatives, **VOMe** and **VNH_2_**, have been shown to form 2:2 complexes with CB[8] resulting in homodimers with similar binding free energies.^33^ As demonstrated by the ^1^H NMR data in Figure 2 b, an equimolar mixture of these two complexes instantaneously produce a statistical distribution of three species in solution, including the two original homodimers (with signals identical to those in Figure 2 a,c) and a third species corresponding to the heterodimer complex. An additional set of peaks emerges for each proton of **VOMe**, **VNH_2_** as well as CB[8], corresponding to the newly formed heterodimer. Integration analysis (e.g. methyl protons at *δ*=3.6 ppm) displays a discrete binomial distribution for the three species with a [AA]:[AB]:[BB] ratio of 1:2:1, suggesting that **VOMe** and **VNH_2_** are randomly associated with each other to form dimers with CB[8]. The equilibrium constant *K* ([Eq. (1)]; Figure 2 g)^41^ for this statistical exchange is calculated to be 1.

(1) $$K=\frac{{\left(\left[\mathrm{AB}\right]/2\right)}^{2}}{\left[\mathrm{AA}][\mathrm{BB}\right]}$$

**Figure 1:**
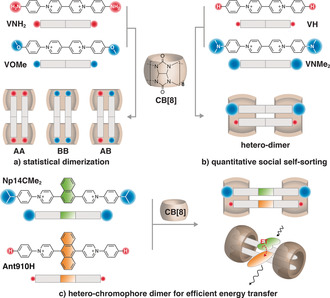
a) Statistical dimerization with the coexistence of three species (AA, BB, and AB). b) Quantitative supramolecular heterodimerization is realized through well‐designed shape complementarity between end‐groups. c) Pure hetero‐chromophore dimers with efficient energy transfer generated from the same species. The Cl^−^ counterions are omitted for clarity.

**Figure 2:**
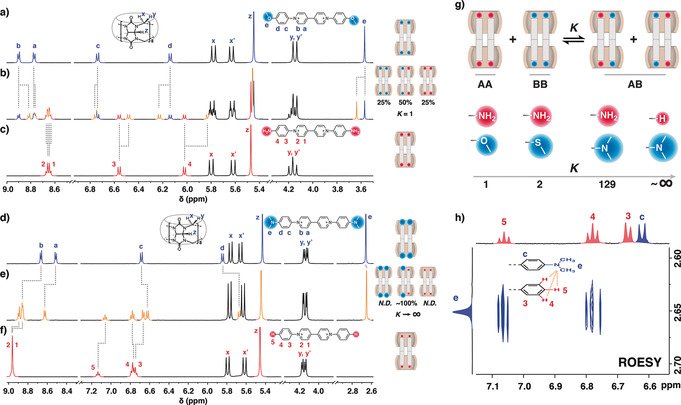
^1^H NMR spectra of CB[8]‐directed homodimers of a) **VOMe**, c) **VNH_2_**, d) **VNMe_2_**, f) **VH**, and the equilibrium products of b) a 50/50 mixture of **VOMe** and **VNH_2_** homodimers as well as e) a 50/50 mixture of **VNMe_2_** and **VH** homodimers. g) The equilibrium constant *K* obtained from integration analysis displays a correlation with the shape complementarity between end‐groups. h) ROESY NMR spectrum of **VNMe_2_** and **VH** homodimer mixture shows a clear cross‐correlation between the *N*,*N*‐dimethylamino substituent of **VNMe_2_** and the phenyl group of **VH**. The Cl^−^ counterions are omitted for clarity.

One would expect an extremely large value of *K* (ideally infinity) upon quantitative heterodimer formation. A large *K* value requires highly selective self‐sorting between A and B with suppression of self‐recognition (AA and BB)^55^ and a substantial elevation of self‐discrimination (AB)^56^ at equilibrium. Würthner et al. suggested that appropriately designed shape complementarity combined with short‐range attractions may provide an effective route to hetero‐aggregates through self‐sorting.^57^, ^58^ We have recently shown that the homodimer stacking of BAP derivatives with CB[8] is susceptible to the bulkiness of substituents at both ends of the complex.^34^, ^36^ Large substituents lead to stacking with a large slippage between chromophores along the long‐molecule axis on account of steric clash.^34^ We therefore posited that a quantitative heterodimer AB could be realized through a careful selection of a large end‐group for A and a complementary small end‐group for B. Thus in a 50/50 mixture of A and B, the self‐recognition product AA is not favored on account of designed steric crowding, while the preferred self‐discrimination product AB will be significantly enhanced by shape‐complementarity of the big and small end‐groups (Figure 1 b).

Replacing the methoxy group in **VOMe** with an *N*,*N*‐dimethylamino (NMe_2_) substituent yields another BAP derivative, **VNMe_2_**, bearing a bulkier end‐group. A 50/50 mixture of the homodimers **VNMe_2_** and **VNH_2_** displays an equilibrium mixture significantly dominated by the heterodimer (92 %) along with trace amounts of homodimers, as shown in Figure S2 (see the Supporting Information), resulting in a *K* value of 129. In addition to increasing the size of one of the end‐groups (i.e. **VNMe_2_**), a reduction to the size of the complementary end‐group (from **VNH_2_** to **VH**; Figure 2 g) should further increase the value of *K*. Figure 2 e displays an equimolar mixture of **VNMe_2_** and **VH** homodimers, which leads solely to the formation of the desired heterospecies. Even using an NMR instrument equipped with a highly sensitive cryoprobe, proton signals from the original homodimers remain undetectable in solution. The 2D ROESY NMR spectrum (Figure 2 h) shows a clear cross‐correlation between the *N*,*N*‐dimethylamino substituent of **VNMe_2_** and the phenyl group of **VH**. This cross‐correlation indicates the close proximity of the two guest molecules and explicitly confirms formation of heterodimers, which simultaneously contain one **VNMe_2_** and one **VH** complexed with two CB[8] macrocycles.

We then applied our strategy to generate heterodimers using extended BAP derivatives to obtain discrete chromophore‐coupled dimers. The extended BAP derivative **Np14NMe_2_**, containing a 1,4‐naphthyl core and bulky dimethylamino end‐groups, can be mixed with **Np14H** and CB[8] to quantitatively produce the heterodimer product (see Figures S7–S9). While quantitative heterodimer is formed, the use of NMe_2_ as an end‐group led to complete quenching of 1,4‐naphthyl fluorescence upon photoexcitation on account of photoinduced electron transfer. This undesired quenching was readily overcome by exchanging NMe_2_ for an isopropyl moiety (CMe_2_), yielding **Np14CMe_2_**. A previous photophysical study^36^ showed that the self‐recognition product of **Np14CMe_2_** with CB[8] (i.e. homodimer, AA) is substantially suppressed because of its bulky end‐groups, thus suggesting **Np14CMe_2_** may be a better candidate to generate pure heterodimers with **Np14H**. Indeed, an equimolar mixture of **Np14CMe_2_** and **Np14H** with two equivalents of CB[8] instantaneously leads a quantitative self‐sorting of heterodimers at equilibrium (see Figures S10–S13).

Strictly speaking, the complex of **Np14CMe_2_** and **Np14H** does not represent a hetero‐chromophore dimer as their central cores are identical. Therefore, we synthesized additional extended BAPs, **Ant910H** and **Ant910CMe_2_**, using a 9,10‐anthracenyl group as the central chromophore, which is also one aryl unit in width. Figure 3 b shows the proton spectrum for the equilibrium mixture of **Np14CMe_2_**, **Ant910H**, and CB[8] with a molar ratio of 1:1:2. Only one set of CB[8] protons is observed with a splitting pattern typical for 2:2 complexation, which is further confirmed by a diffusion constant of 1.98×10^−10^ m^2^ s^−1^ measured by DOSY NMR spectroscopy, exhibiting a typical value for a complex containing two CB[8] macrocycles (see Table S1).^34^, ^36^, ^59^ Variable‐temperature NMR measurements (see Figure S15) display a gradual signal broadening and eventual coalescence of the pyridinium protons in **Ant910H** as the temperature increases from 278 to 317 K. This temperature‐dependent behavior corresponds to restricted intra‐complex rotation of the central anthracenyl moiety with an intermediate rate related to the NMR timescale.^18^, ^36^ NOESY NMR offers definitive evidence for proximal stacking the two different chromophore moieties. In particular, clear correlations between the isopropyl group of **Np14CMe_2_** and the phenyl group of **Ant910H** (Figure 3 d) confirm successful formation of CB[8]‐directed hetero‐chromophore complexation. The desired heterospecies was observed by direct injection of the complex solution into ESI‐MS using nanospray ionization, showing complex ion peaks with four positive charges (*z=*4) and a *m*/*z* ratio centered around 916 (Figure 3 e), perfectly consistent with the *m*/*z* for **[Np14CMe_2_⋅Ant910H⋅CB[8]_2_]^4+^**. Importantly, swapping the end‐groups of the two extended BAPs (i.e. **Np14H** and **Ant910CMe_2_**) also leads to the desired complementary heterodimer (see Figures S19–S23).

**Figure 3:**
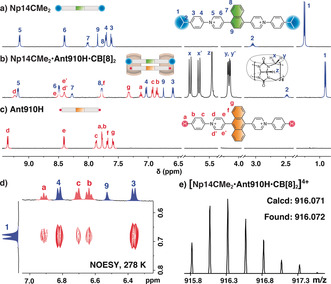
^1^H NMR spectra of a) **Np14CMe_2_**, c) **Ant910H**, and b) their equimolar mixture with two equivalents of CB[8], which displays a proximal stacking between the isopropyl moiety of **Np14CMe_2_** and the phenyl group of **Ant910H** demonstrated by 2D NOESY NMR at 278 K (d). All protons are unambigiously assigned through 2D COSY and NOESY NMR (see Figures S16 and S17). e) Complex ion peaks with four charges (*z=*4) and a *m*/*z* centered around 916 in the ESI‐MS confirm the formation of **[Np14CMe_2_⋅Ant910H⋅CB[8]_2_]^4+^** heterodimer. The Cl^−^ counterions are omitted for clarity.

NOESY NMR data (see Figure S17) indicates that 1,4‐naphthyl and 9,10‐anthracenyl chromophores are forced to stack on top of each other with a spacing of approximately 4 Å, as limited by the CB[8] cavity.^22^, ^23^, ^60^ Moreover, the pseudostatic nature of the discrete CB[8] dimer combined with its observed restricted intracomplex motions gives rise to a self‐assembled system poised to exploit long‐lived hetero‐chromophore coupling with efficient energy transfer.

Energy transfer in a supramolecular system can be readily investigated through steady‐state spectroscopic measurements.^61^, ^62^ For instance, the complexation of one equivalent of either **Np14CMe_2_** or **Ant910H** with two equivalents of CB[7] generates discrete monomers in aqueous solution (Figure 4 a; see Figure S24).^34^, ^36^ Their steady‐state spectra in Figure 4 c shows that the superposition (the mathematical “mixture”) of both monomers’ spectra (Figure 4 b) resulting in absoroption and emission profiles identical to those recorded for their 50/50 physical mixture. This result indicates that the two discrete monomer complexes behave independently in a mixed solution and that no energy transfer takes place between naphthyl and anthracenyl chromophores upon photoexcitation.

**Figure 4:**
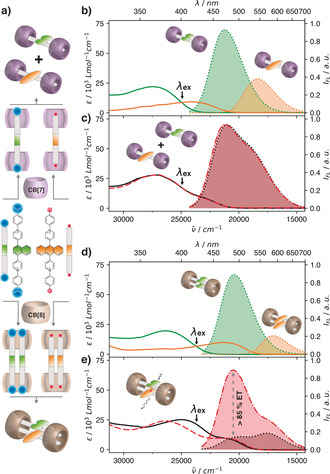
a) Discrete monomers and dimers of **Np14CMe_2_** or **Ant910H** can be generated through complexation with CB[7] and CB[8], respectively. b–e) Steady‐state spectra for monomeric or dimeric species of **Np14CMe_2_** (green) and **Ant910H** (orange) and their physical (black) and mathematical mixtures (red, dashed). All solutions were excited at their isosbestic point (401 nm for CB[7] and 420 nm for CB[8]) to ensure an equal distribution of photons into both chromophores. The energy‐transfer efficiency was estimated by comparing the naphthyl emission at 485 nm between the mathematical and physical mixtures. Absorption: solid lines; emission: dotted lines with filled color. The Cl^−^ counterions are omitted for clarity.

A 50/50 physical mixture of CB[8]‐mediated **Np14CMe_2_** and **Ant910H** homodimers yields hetero‐chromophore dimers quantitatively, whose emission spectrum (Figure 4 e) is significantly different from that of their mathematical mixture. The emission intensity around 490 nm (from 1,4‐naphthyl chromophore) is substantially reduced in the heterodimers, suggesting the existence of a fast non‐radiative pathway to quench the photoexcitated naphthyl chromophore. In contrast, emission of the 9,10‐anthracenyl chromophore at 650 nm mainly stems from the excitation around 400 nm (see Figure S25), which is identical to the absorption band of the 1,4‐naphthyl moiety. This suggests that non‐radiative energy transfer takes place from the photoexcited naphthyl moiety to its neighboring anthracenyl group in the discrete heterodimer with an extremely high efficiency estimated to be greater than 85 %. The nature of this observed energy transfer (short‐ and/or long‐ranged) is of great importance for the design of future systems, which is currently being investigated with time‐resolved techniques.

In summary, we developed a straightforward supramolecular strategy based on CB[8]‐directed dimerization to prepare hetero‐chromophore dimers by simply mixing two precusors. The challenge of quantitatively forming heterodimers without other equilibrium by‐products is overcome through self‐sorting favored by the introduction of designed shape‐complementary moieties. The resulting pseudostatic heterodimers enables strong, long‐lived coupling between chromophores, which leads to efficient energy transfer upon photoexcitation. Using this supramolecular strategy, which is superisingly flexible and validated by five cases here, a broad range of discrete chromophore dimers can be readily prepared, beneficial to further investigation and understanding of exciton coupling. Moreover, the noncovalent nature of these assemblies facilitates optimal overlap between desired chromphores, providing alternative tectons for the development of high‐performance optoelectronic materials through self‐assembly.

## Conflict of interest

The authors declare no conflict of interest.

## Supporting information

As a service to our authors and readers, this journal provides supporting information supplied by the authors. Such materials are peer reviewed and may be re‐organized for online delivery, but are not copy‐edited or typeset. Technical support issues arising from supporting information (other than missing files) should be addressed to the authors.

Supplementary
