# Supplementary material for: Quantitative Supramolecular Heterodimerization for Efficient Energy Transfer
Source: Angew Chem Int Ed Engl. 2020 Jul 13;59(37):15963–7. doi: 10.1002/anie.202006530 (PMC7540262; doi:10.1002/anie.202006530)
Supplement: Supplementary file 1 — Supplementary [file ANIE-59-15963-s001.pdf]

Angewandte Chemie
Eine Zeitschrift der Gesellschaft Deutscher Chemiker

Supporting Information

Quantitative Supramolecular Heterodimerization for Efficient Energy Transfer

*Guanglu Wu, Zehuan Huang, and Oren A. Scherman**

anie_202006530_sm_miscellaneous_information.pdf

Table of Contents

SI-1 Materials and methods 1
SI-2 Synthesis and characterization 3
SI-3 Mixture of VNH_2 and VOMe homodimers 7
SI-4 Mixture of VNH_2 and $VNMe_2$ homodimers 7
SI-5 Mixture of VNH_2 and VSMe homodimers 8
SI-6 Mixture of VH and $VNMe_2$ homodimers 9
SI-7 Mixture of Np14H and $Np14NMe_2$ homodimers 10
SI-8 Mixture of Np14H, $Np14CMe_2$, and CB[8] 12
SI-9 Mixture of Ant910H, $Np14CMe_2$, and CB[8] 14
SI-10 Mixture of Np14H, $Ant910CMe_2$, and CB[8] 17
SI-11 Diffusion coefficient of CB[8]-directed dimers 19
SI-12 Complexation of Ant910H and $Np14CMe_2$ with CB[7] 20
SI-13 Excitation spectrum of Ant910H and $Np14CMe_2$ dimers 21
Reference 21

SI-1 Materials and methods

Materials. All materials were purchased from commercial suppliers as follows and used without further purification: 9,10-dibromoanthracene (98%, Sigma-Aldrich), 4-pyridinylboronic acid (90%, Sigma-Aldrich), tetrakis(triphenylphosphine) palladium(0) (99%, Alfa Aesar), 1-chloro-2,4-dinitrobenzene (98%, Alfa Aesar), aniline (99.5%, Sigma-Aldrich), 4-isopropylaniline (99%, Sigma-Aldrich), 2,2-Dimethyl-2-silapentane-5-sulfonate sodium salt (97%, Sigma-Aldrich). All other reagents and solvents were purchased from Sigma-Aldrich (UK) or Fisher Scientific and used as received. Cucurbit[8]uril (CB[8]) and CB[7] were synthesized and purified according to a published procedure[1]. Milli-Q water (18.2 MΩ·cm) was used to prepare all non-deuterated aqueous solutions.

High Pressure Liquid Chromatography (HPLC). HPLC was employed to purify and collect dicationic species by using a 150 x 21.2 mm Phenomenex C18 Kinetic-Evo column with a 5 micron pore size and a 110 Å particle size. A gradient from 5% acetonitrile 95% water to 100% acetonitrile was used with 0.1% TFA.

Nuclear Magnetic Resonance Spectroscopy (NMR). ^{1}H NMR, ^{13}C NMR, COSY, DOSY, ROESY, and NOESY spectra were acquired in heavy water (D_2O) and recorded on a Bruker AVANCE 500 with TCI Cryoprobe system (500 MHz) being controlled by TopSpin2. NOESY experiments were carried out using a standard pulse sequence 'noesygpphpp' with a 2 s relaxation delay and a 1000 ms mixing time. The concentration of CB[8] or CB[7] deuterated aqueous solution was calibrated by an internal standard: DSS sodium salt. ROESY experiments were carried out using a modified EASY ROESY pulse sequence 'roesyadjsphpr' with a 2 s relaxation delay and 200 ms mixing time.

Variable-Temperature ^{1}H NMR (VT-NMR). ^{1}H NMR spectra with variable temperature were recorded by a Bruker AVANCE 500 with TCI Cryoprobe system (500 MHz).

Diffusion Ordered Spectroscopy (DOSY). The ^{1}H DOSY experiments were carried out using a modified version of the Bruker sequence ledbpgp2s involving, typically, 32 scans over 16 steps of gradient variation from 10% to 80% of the maximum gradient. Diffusion coefficients were evaluated in Dynamic Centre (a standard Bruker software) and determined by fitting the intensity decays according to the following equation:

$$I = I_o\, e^{[-D\gamma^2 g^2 \delta^2 (\Delta - \delta/3)]}$$

where I and I_o represent the signal intensities in the presence and absence of gradient pulses respectively, D is the diffusion coefficient, $\boldsymbol{\gamma}$ = 26753 rad/s/Gauss is the ^{1}H gyromagnetic ratio, δ = 2.4 ms is duration of the gradient pulse, Δ = 100 ms is the total diffusion time and g is the applied gradient strength. The Monte Carlo simulation method was used for the error estimation of fitting parameters with a confidence level of 95%.

UV/Vis Spectroscopy and Fluorescence Spectroscopy. UV/Vis and fluorescence spectra were recorded on a Varian Cary 400 UV/Vis spectrophotometer and Varian Cary Eclipse fluorescence spectrophotometer, respectively, using a Hellma 114F-QS cuvette with 10x4 mm path length at 298 K.

Electrospray Ionization Mass Spectrometry (ESI-MS). ESI-MS spectra were acquired on a Thermo Fisher Q Exactive Orbitrap mass spectrometer with a nanospraying ion source. Positive mode was chosen for all the experiments with the working temperature of 320 °C and the capillary voltage of 1.5 kV. All the sample solutions were prepared in pure water.

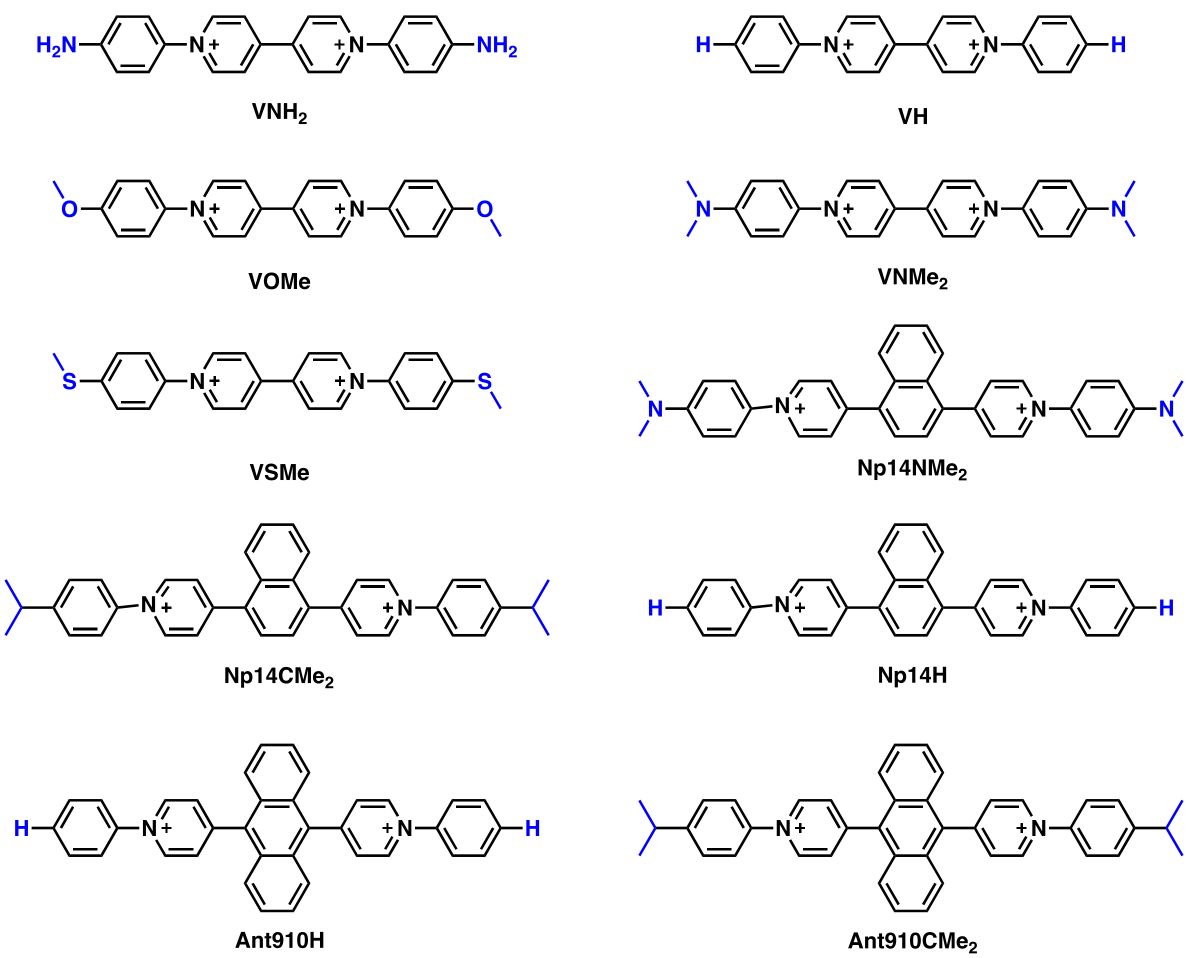

Scheme S1. Molecules related to this work (Counter anion is Cl^-).

SI-2 Synthesis and characterization

As exemplified by **Ant910X (X= H, CMe₂)** in Scheme S2, a general synthesis of extended bis(*N*-arylpyridinium) derivatives in this work starts with Suzuki-Miyaura cross-coupling of two pyridin-4-yl groups onto the fluorophore core, followed by the transformation of the pryidin-4-yl groups into arylpyridinium salts through a Zincke reaction.[2] The synthesis of **VX (X= H, NH_2, OMe, SMe, NMe_2)**,[3-5] **Ant910DNB**,[6] and **Np14X (X = DNB, H, NMe_2)** were reported in our previous work.[6-7] The final Zincke reactions are pseudo-quantitative, generally resulting in an isolation yield larger than 80 % even in a 10 mg scale reaction.

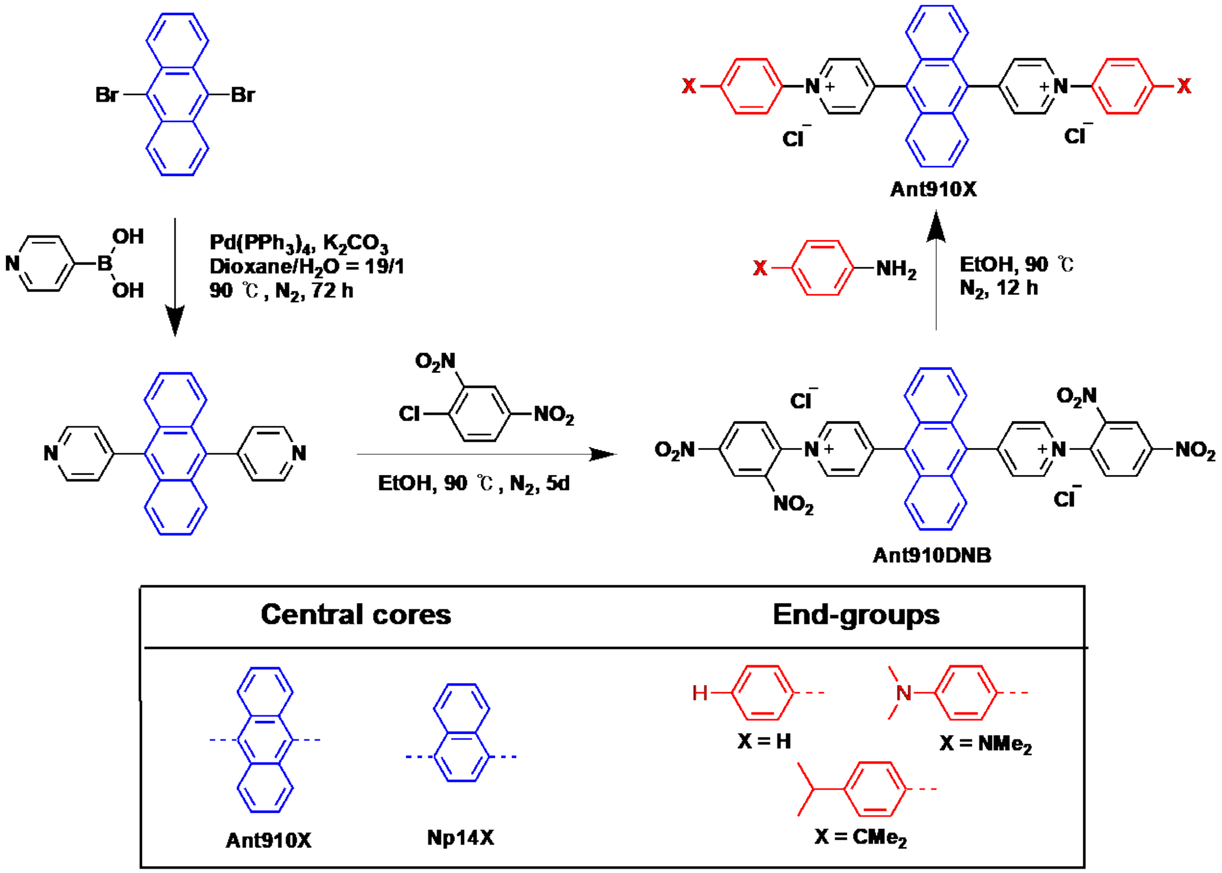

Scheme S2. Synthetic route of **Ant910X**.

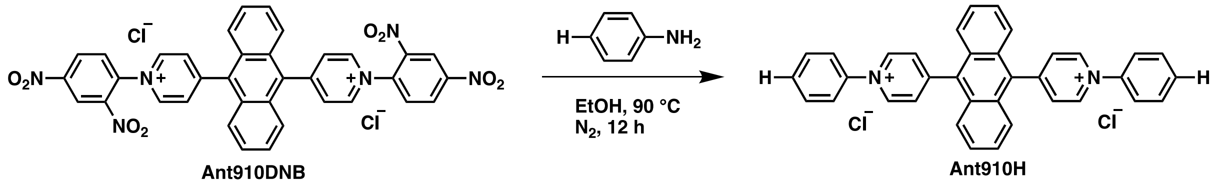

4,4'-(anthracene-9,10-diyl)bis(1-phenylpyridin-1-ium) chloride (Ant910H). Aniline (30 µL, excess) and 4,4'-(anthracene-9,10-diyl)bis(1-(2,4-dinitrophenyl)pyridin-1-ium) chloride (**Ant910DNB**, 6 mg, 8.6 µmol) were refluxed in ethanol (10 mL) at 90 °C for 12 h under nitrogen atmosphere. After removing solvent under reduced pressure, the solid was re-dissolved in MeOH (1 mL) and was added dropwise into diethyl ether (30 mL) to precipitate products. The suspension was then centrifuged at 8000 rpm for 10 mins at 4 °C and the supernatant was decanted. The centrifugation/decanting cycle was repeated another two times in order to wash off excess of aniline. Drying in vacuum oven gave 4 mg of orange solid in 89 % yield. ^{1}H NMR (500 MHz, D_2O) δ (ppm): δ 9.35 (d, J = 6.7 Hz, 4H), 8.41 (d, J = 6.5 Hz, 4H), 7.87 (dd, J = 6.8, 2.8 Hz, 4H), 7.80 – 7.74 (m, 6H), 7.68 (dd, J = 6.8, 3.2 Hz, 4H), 7.59 (dd, J = 6.9, 3.2 Hz, 4H). ^{13}C NMR (126 MHz, D_2O) δ (ppm): 157.72, 144.50, 132.60, 131.72, 131.10, 130.97, 130.62, 128.13, 127.47, 125.32, 124.12. LCMS: m/z $[M\text{-}2Cl]^{2+}$ calcd. for $C_{36}H_{26}N_2^{2+}$: 243.1, found: 243.4.

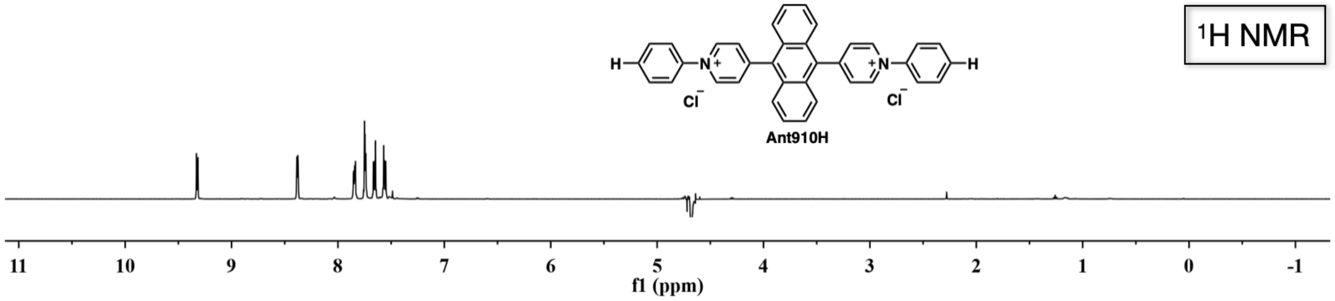

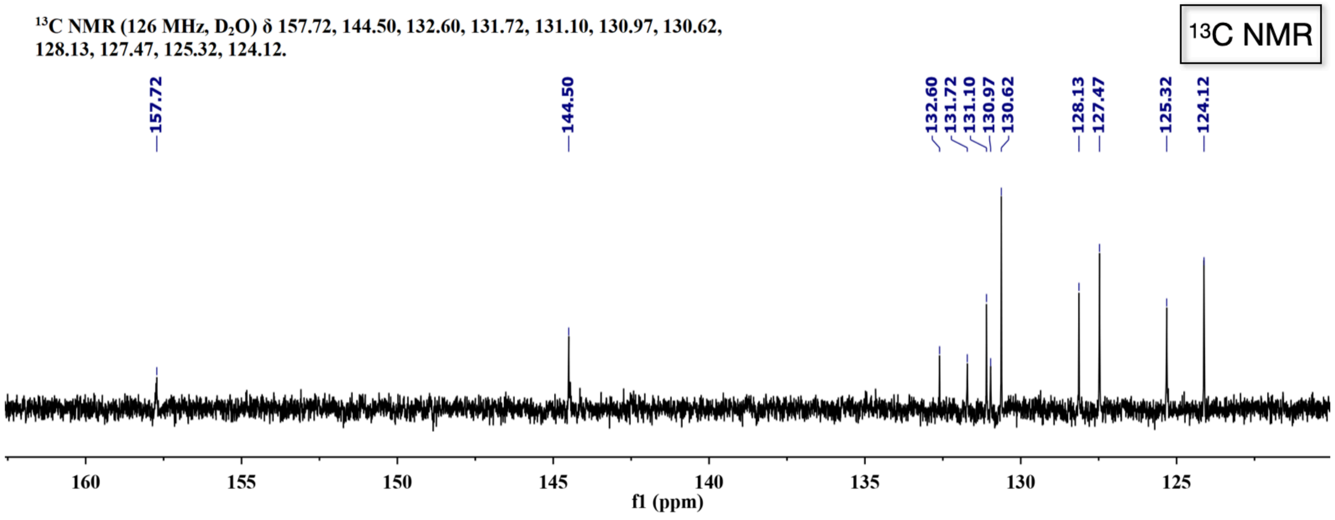

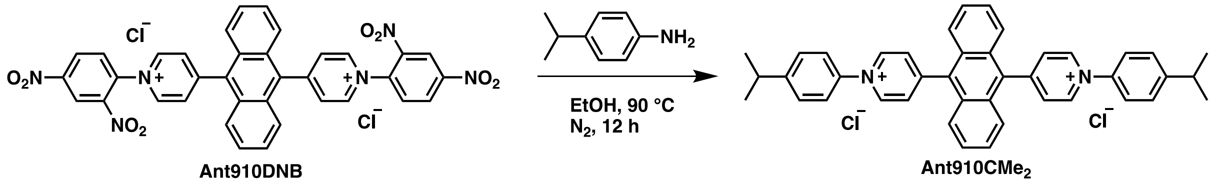

4,4'-(anthracene-9,10-diyl)bis(1-(4-isopropylphenyl)pyridin-1-ium) chloride (Ant910CMe$_2$). **Ant910CMe$_2$** was prepared following the same synthetic route as that of **Ant910H** except using 4-isopropylaniline instead of aniline as the starting material. Yield 83 %, 15 mg. ^{1}H NMR (500 MHz, D_2O) δ (ppm): 9.31 (d, J = 6.3 Hz, 4H), 8.38 (d, J = 6.4 Hz, 4H), 7.79 (d, J = 8.3 Hz, 4H), 7.70 – 7.63 (m, 8H), 7.58 (dd, J = 6.9, 3.2 Hz, 4H), 3.08 (p, J = 7.0 Hz, 2H), 1.28 (d, J = 6.9 Hz, 12H). ^{13}C NMR (126 MHz, MeOD) δ (ppm): 157.73, 153.30, 144.84, 140.94, 132.86, 130.81, 128.49, 128.44, 127.35, 125.38, 124.13, 33.88, 22.75. LCMS: m/z [M-2Cl]$^{2+}$ calcd. for $C_{42}H_{38}N_2^{2+}$: 285.1, found: 285.5.

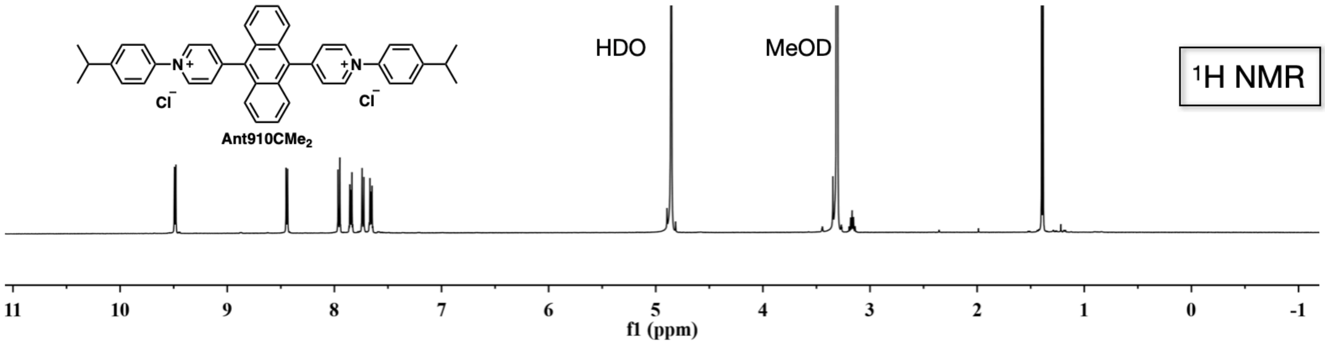

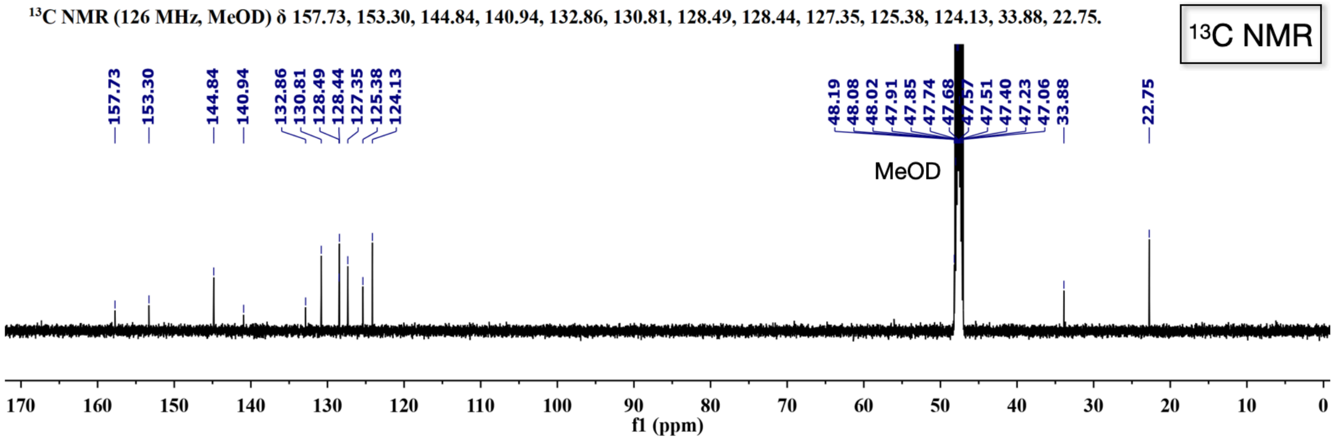

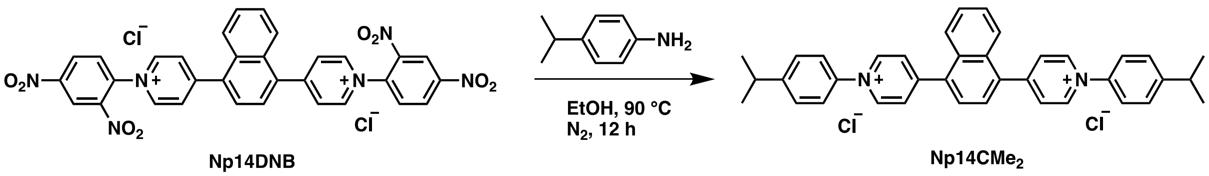

4,4'-(naphthalene-1,4-diyl)bis(1-(4-isopropylphenyl)pyridin-1-ium) chloride ($Np14CMe_2$). **$Np14CMe_2$** was prepared following the same synthetic route as that of **$Ant910CMe_2$** except using **Np14DNB** instead of **Ant910DNB** as the starting material. Yield 87 %, 10 mg. ^1H NMR (500 MHz, D_2O) δ (ppm): 9.24 (d, J = 6.9 Hz, 4H), 8.49 (d, J = 6.8 Hz, 4H), 8.10 (dd, J = 6.5, 3.3 Hz, 2H), 7.93 (s, 2H), 7.83 – 7.77 (m, 6H), 7.70 (d, J = 8.6 Hz, 4H), 3.14 (p, J = 6.9 Hz, 2H), 1.34 (d, J = 6.9 Hz, 12H). ^{13}C NMR (126 MHz, D_2O) δ (ppm): 157.53, 153.35, 143.95, 140.23, 136.45, 130.12, 129.05, 128.57, 128.49, 127.69, 125.11, 123.87, 33.42, 22.98. LCMS: m/z $[M-2Cl]^{2+}$ calcd. for $C_{38}H_{36}N_2^{2+}$: 260.1, found: 260.4.

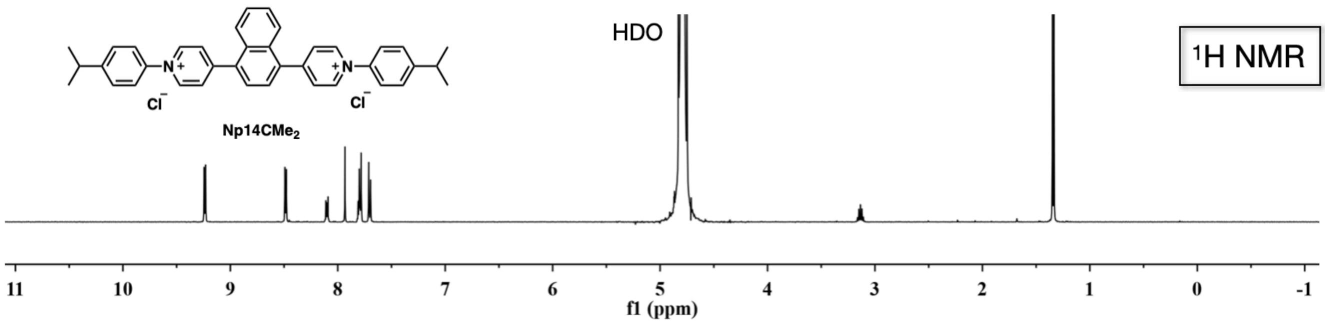

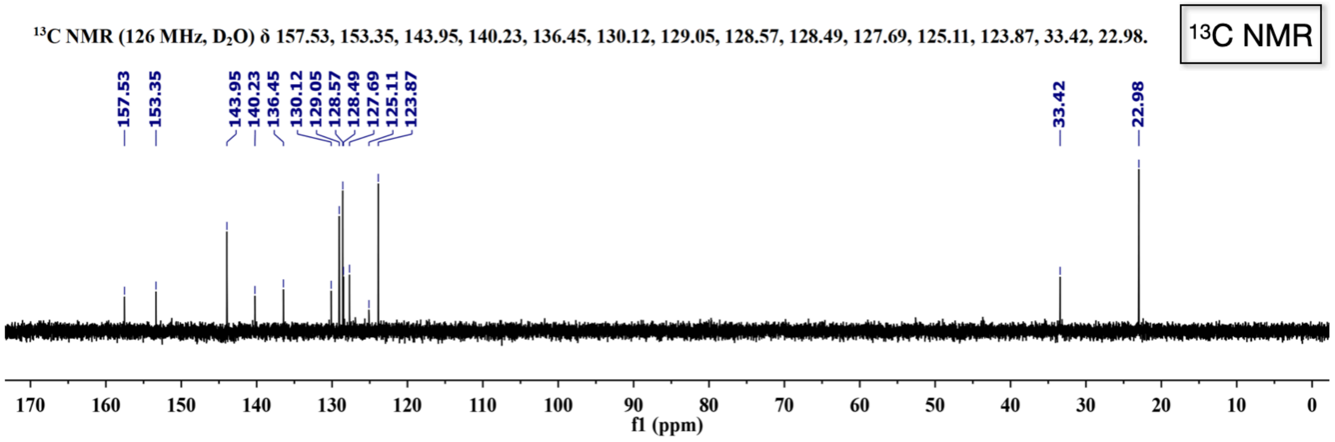

SI-3 Mixture of VNH2 and VOMe homodimers

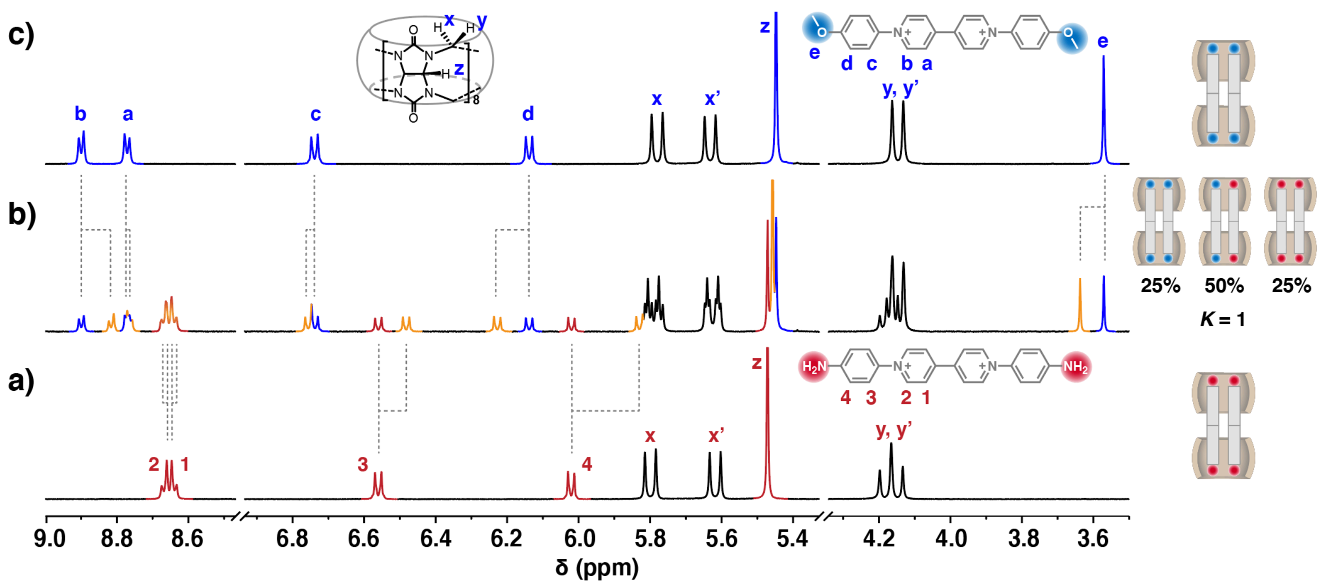

Figure S1. ^{1}H NMR spectra of a) **$(VNH_2)_2$•CB[8]$_2$**, c) **$(VOMe)_2$•CB[8]$_2$**, and b) the equilibrium products of a 50/50 mixture of **$(VNH_2)_2$•CB[8]$_2$** and **$(VOMe)_2$•CB[8]$_2$** in D_2O at 298 K. The K value was calculated according to Eq. 1 in the manuscript, where relative concentration ratios of equilibrium products ([AA], [BB], and [AB]) were quantified from their proton integrations in the ^{1}H NMR spectra.

SI-4 Mixture of VNH2 and VNMe2 homodimers

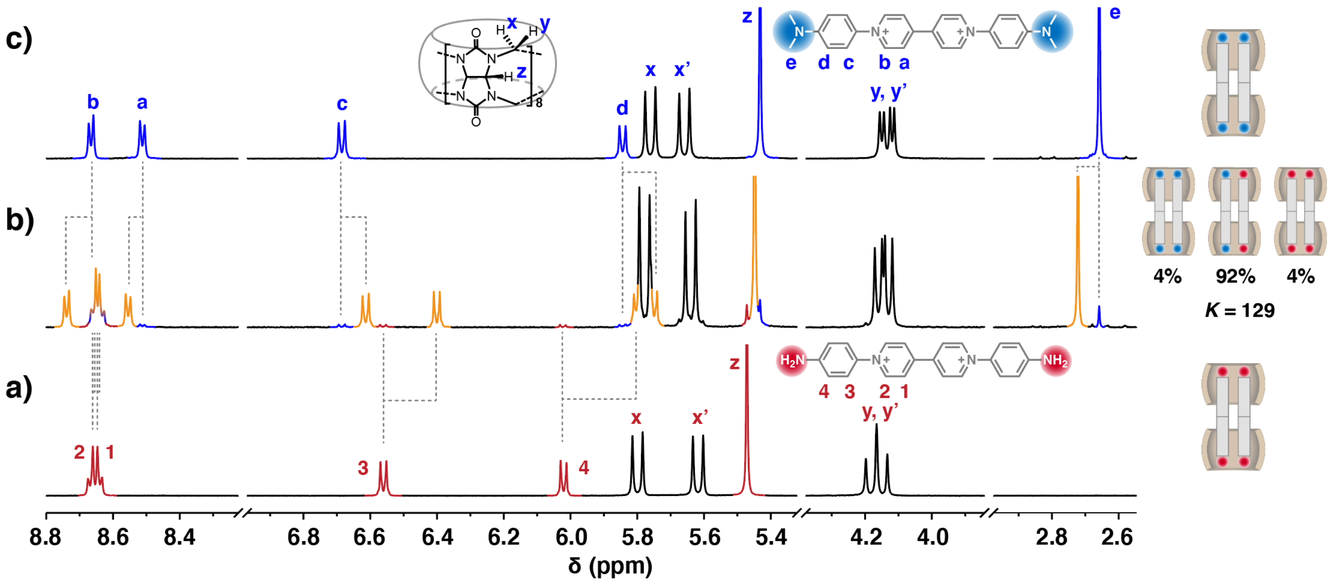

Figure S2. ^{1}H NMR spectra of a) **$(VNH_2)_2$•CB[8]$_2$**, c) **$(VNMe_2)_2$•CB[8]$_2$**, and b) the equilibrium products of a 50/50 mixture of **$(VNH_2)_2$•CB[8]$_2$** and **$(VNMe_2)_2$•CB[8]$_2$** in D_2O at 298 K. The K value was calculated according to Eq. 1 in the manuscript, where relative concentration ratios of equilibrium products ([AA], [BB], and [AB]) were quantified from their proton integrations in the ^{1}H NMR spectra.

SI-5 Mixture of VNH2 and VSMe homodimers

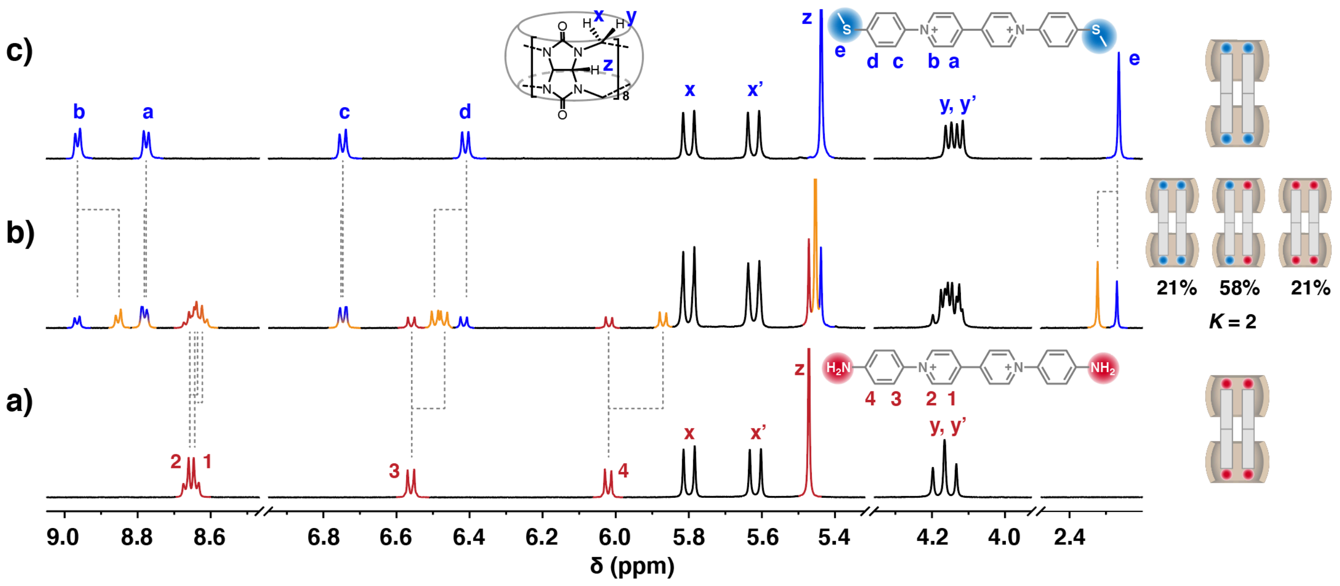

Figure S3. ^{1}H NMR spectra of a) **(VNH2)2•CB[8]2**, c) **(VSMe)2•CB[8]2**, and b) the equilibrium products of a 50/50 mixture of **(VNH2)2•CB[8]2** and **(VSMe)2•CB[8]2** in D2O at 298 K. The K value was calculated according to Eq. 1 in the manuscript, where relative concentration ratios of equilibrium products ([AA], [BB], and [AB]) were quantified from their proton integrations in the ^{1}H NMR spectra.

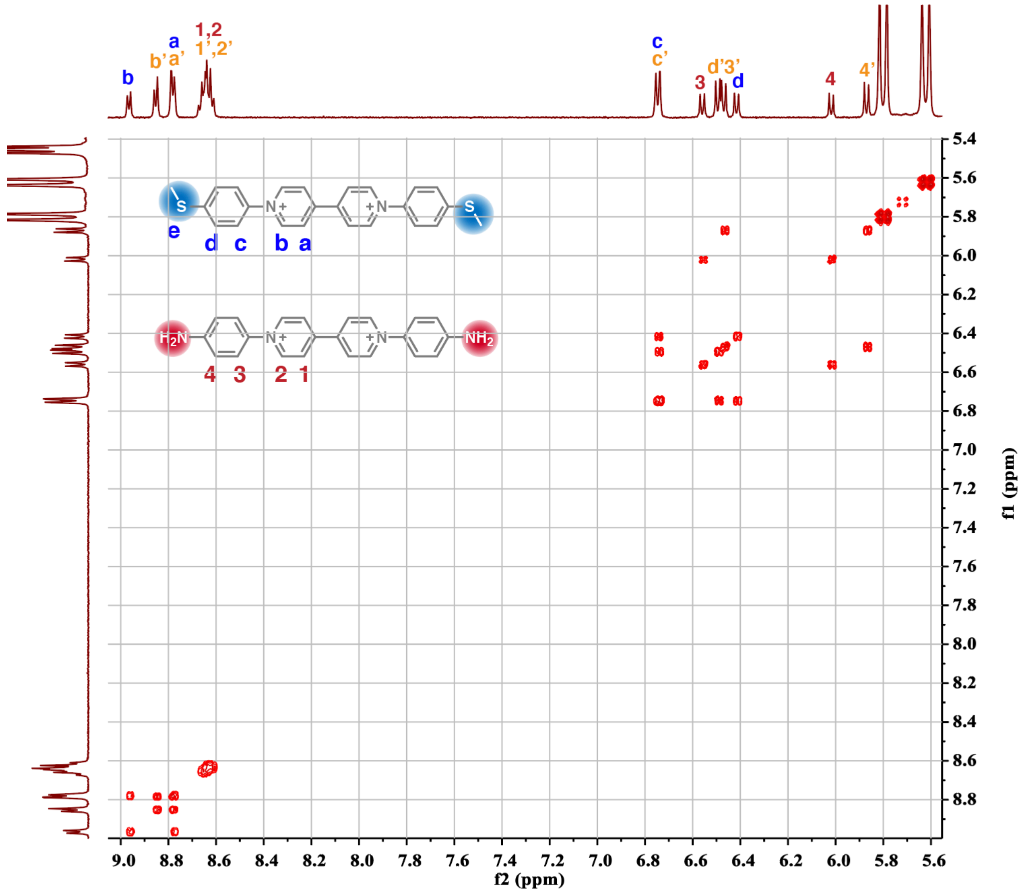

Figure S4. COSY spectrum of the equilibrium products of a 50/50 mixture of **(VNH2)2•CB[8]2** and **(VSMe)2•CB[8]2** in D2O at 298 K, which is carried out in order to assign protons correctly.

SI-6 Mixture of VH and VNMe2 homodimers

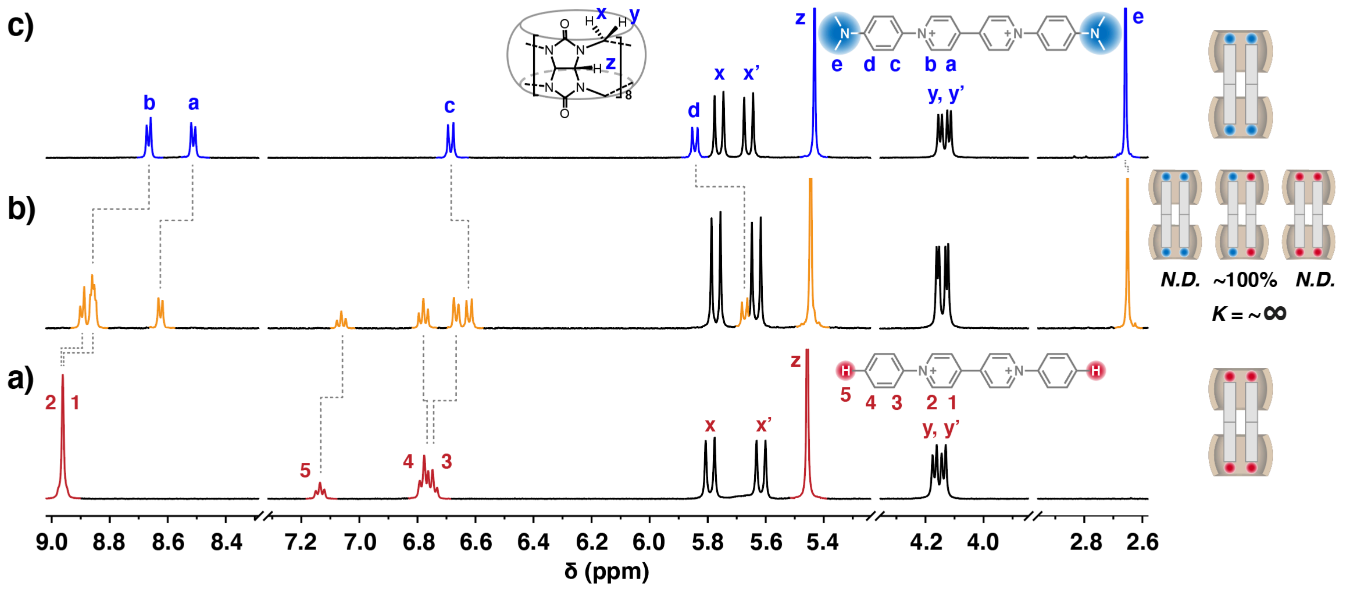

Figure S5. ^{1}H NMR spectra of a) **(VH)$_2$•CB[8]$_2$**, c) **(VNMe$_2$)$_2$•CB[8]$_2$**, and b) the equilibrium products of a 50/50 mixture of **(VH)$_2$•CB[8]$_2$** and **(VNMe$_2$)$_2$•CB[8]$_2$** in D_2O at 298 K. The K value was calculated according to Eq. 1 in the manuscript, where relative concentration ratios of equilibrium products ([AA], [BB], and [AB]) were quantified from their proton integrations in the ^{1}H NMR spectra.

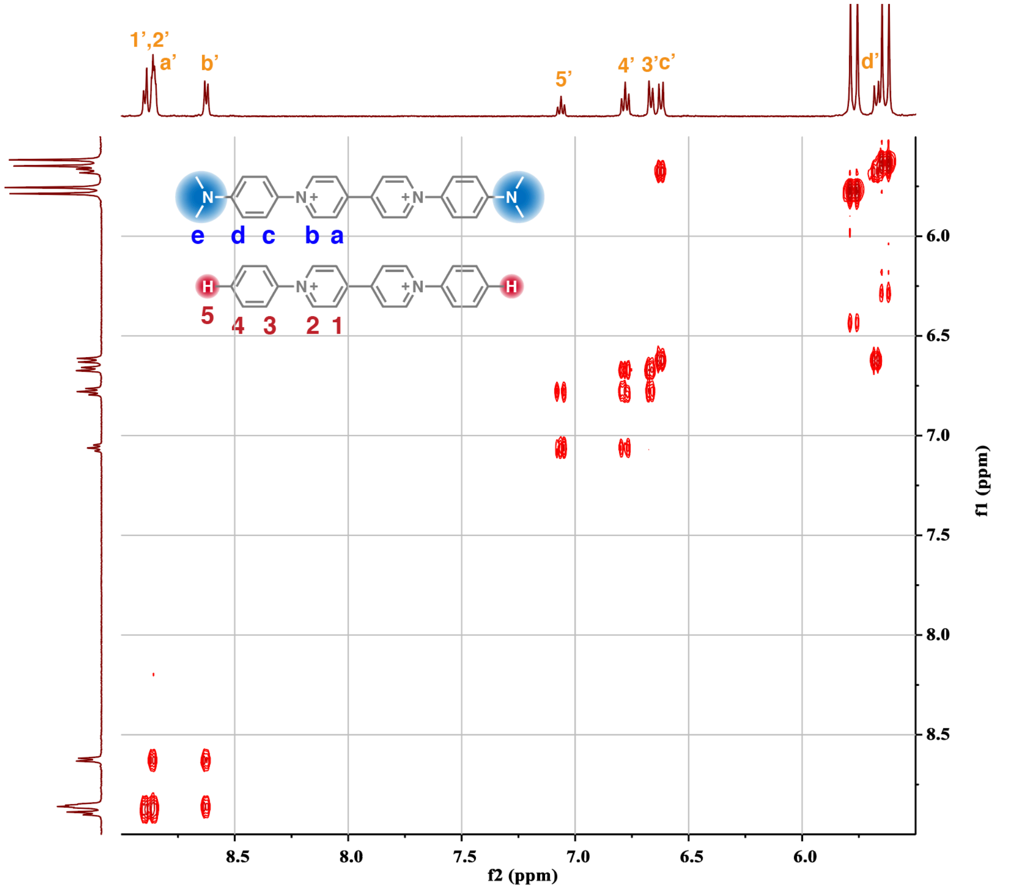

Figure S6. COSY spectrum of **VH• VNMe$_2$•CB[8]$_2$** heterodimer in D_2O at 298 K.

.

SI-7 Mixture of Np14H and Np14NMe₂ homodimers

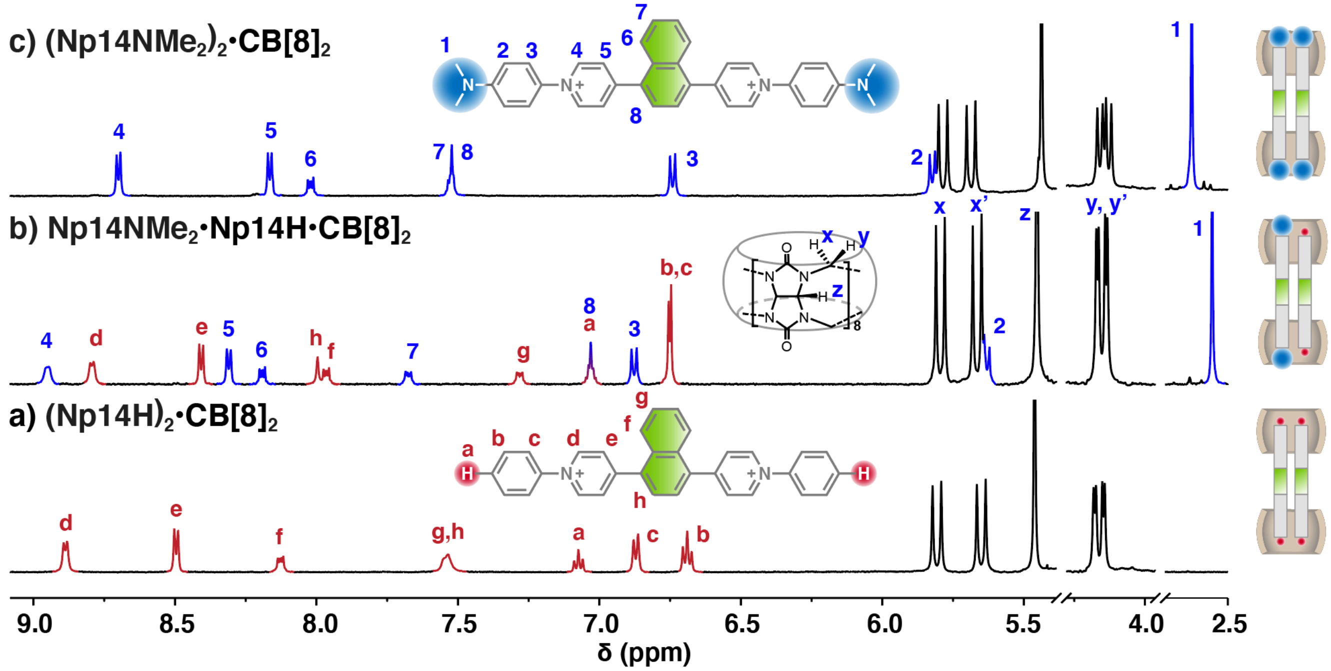

Figure S7. ^{1}H NMR spectra of a) **$(Np14H)_2$•CB[8]$_2$**, c) **$(Np14NMe_2)_2$•CB[8]$_2$**, and b) the equilibrium products of a 50/50 mixture of **$(Np14H)_2$•CB[8]$_2$** and **$(Np14NMe_2)_2$•CB[8]$_2$** in D_2O at 298 K.

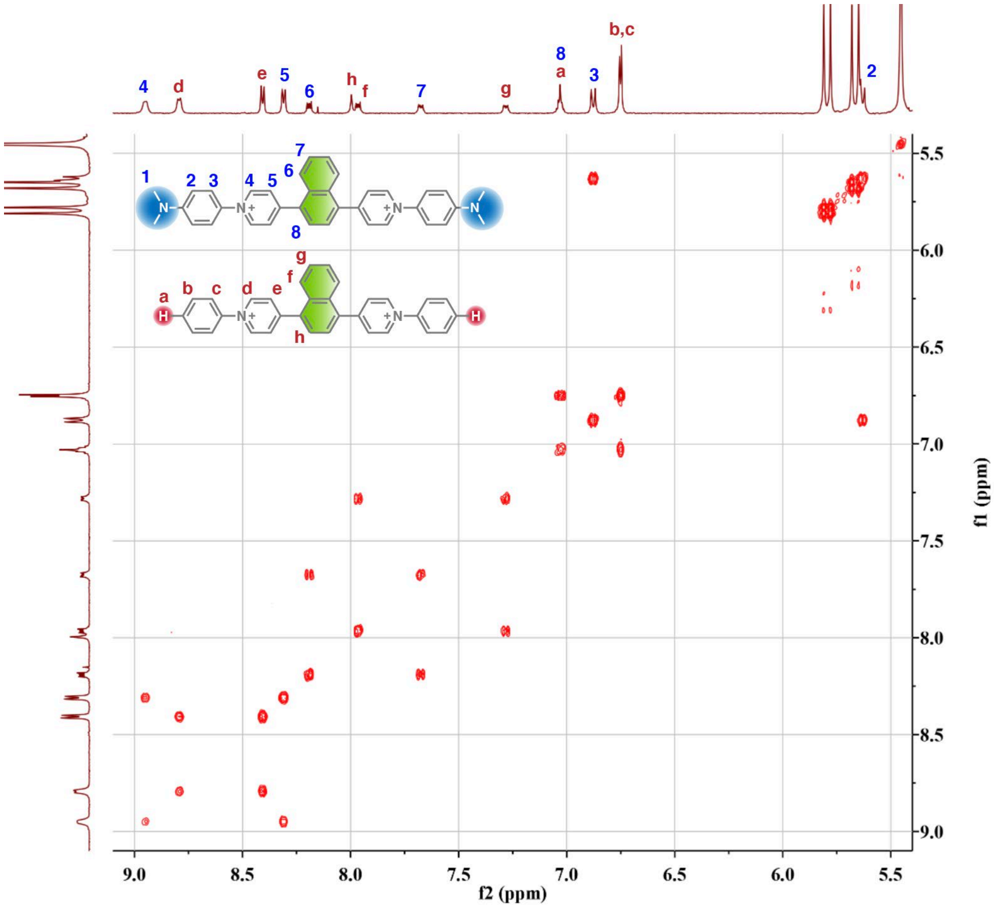

Figure S8. COSY spectrum of **Np14H• Np14NMe$_2$•CB[8]$_2$** heterodimer in D_2O at 298 K.

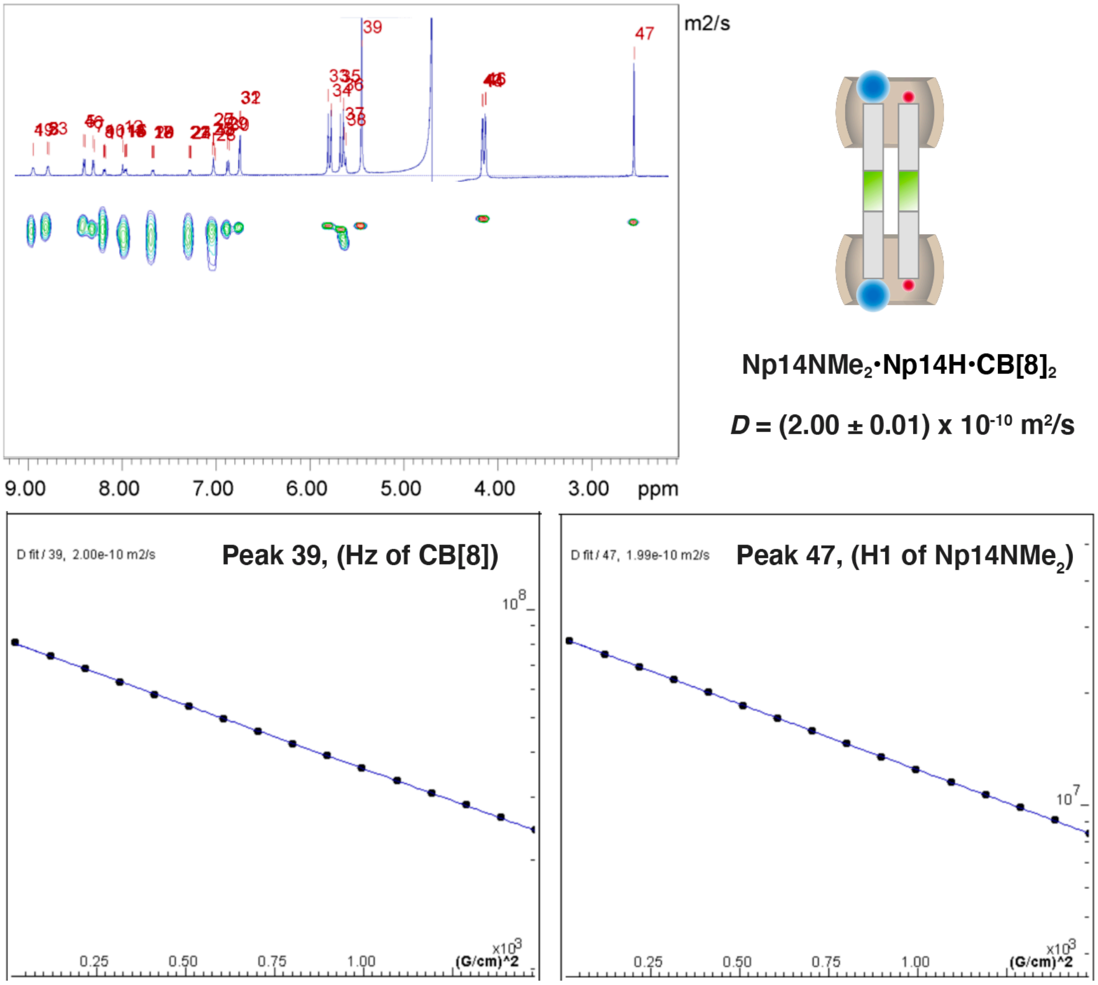

Figure S9. DOSY analysis for **Np14H• Np14NMe$_2$•CB[8]$_2$** heterodimer in D_2O at 298 K, showing one complex with a diffusion coefficient of 2.00 x 10^{-10} m^2/s.

SI-8 Mixture of Np14H, Np14CMe₂, and CB[8]

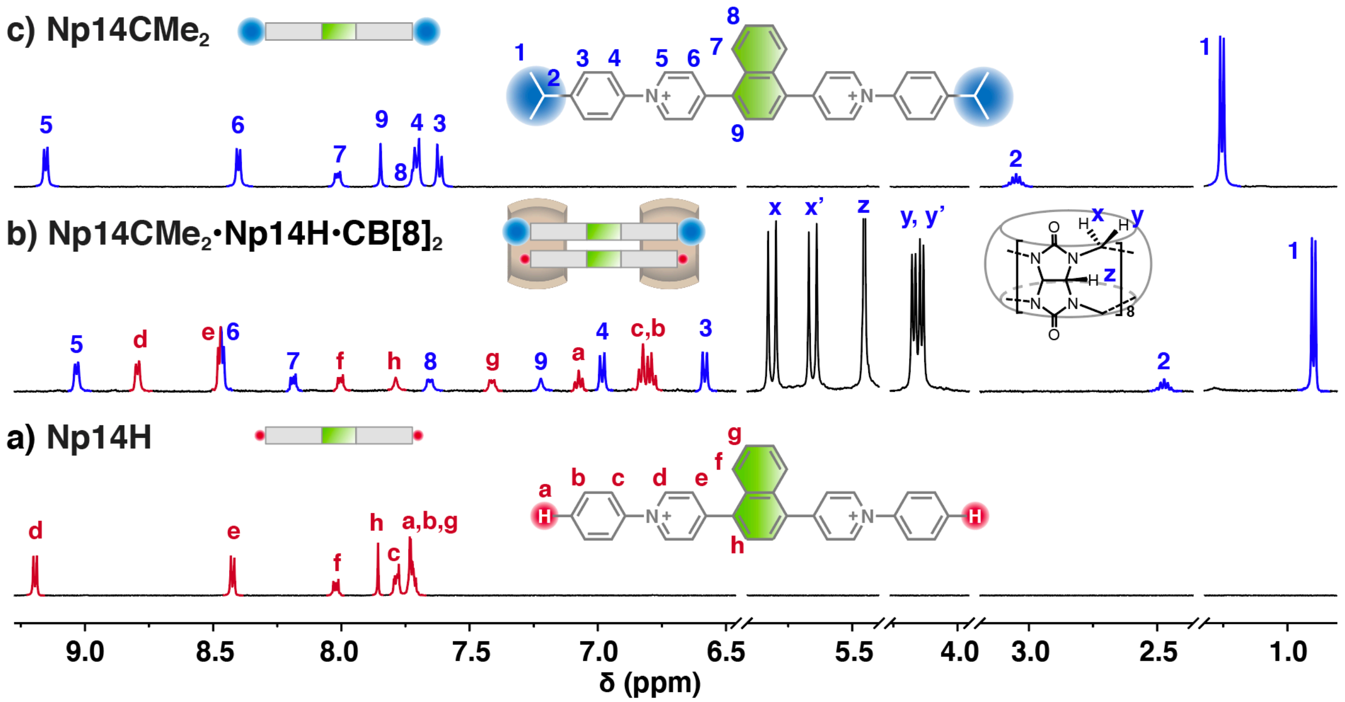

Figure S10. ^{1}H NMR spectra of a) **Np14H**, b) **Np14H• Np14CMe₂•CB[8]₂**, c) **Np14CMe₂** in D_2O at 298 K. Protons are assigned using the COSY and NOESY spectra in Figure S11-12.

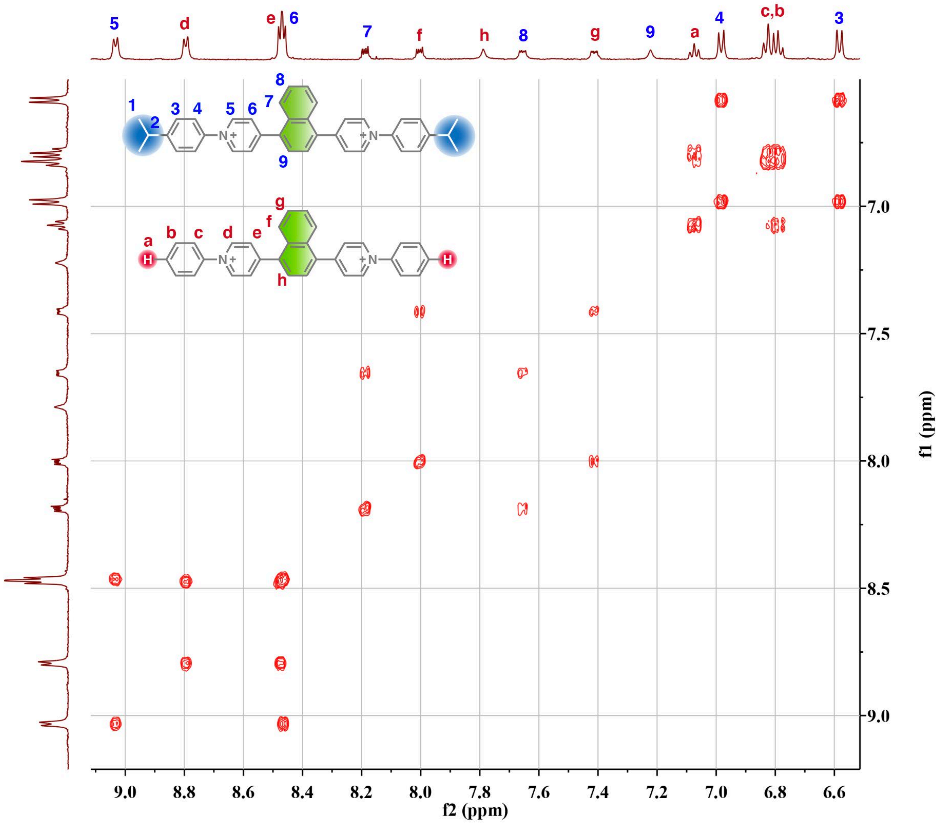

Figure S11. COSY spectrum of **Np14H• Np14CMe₂•CB[8]₂** heterodimer in D_2O at 298 K.

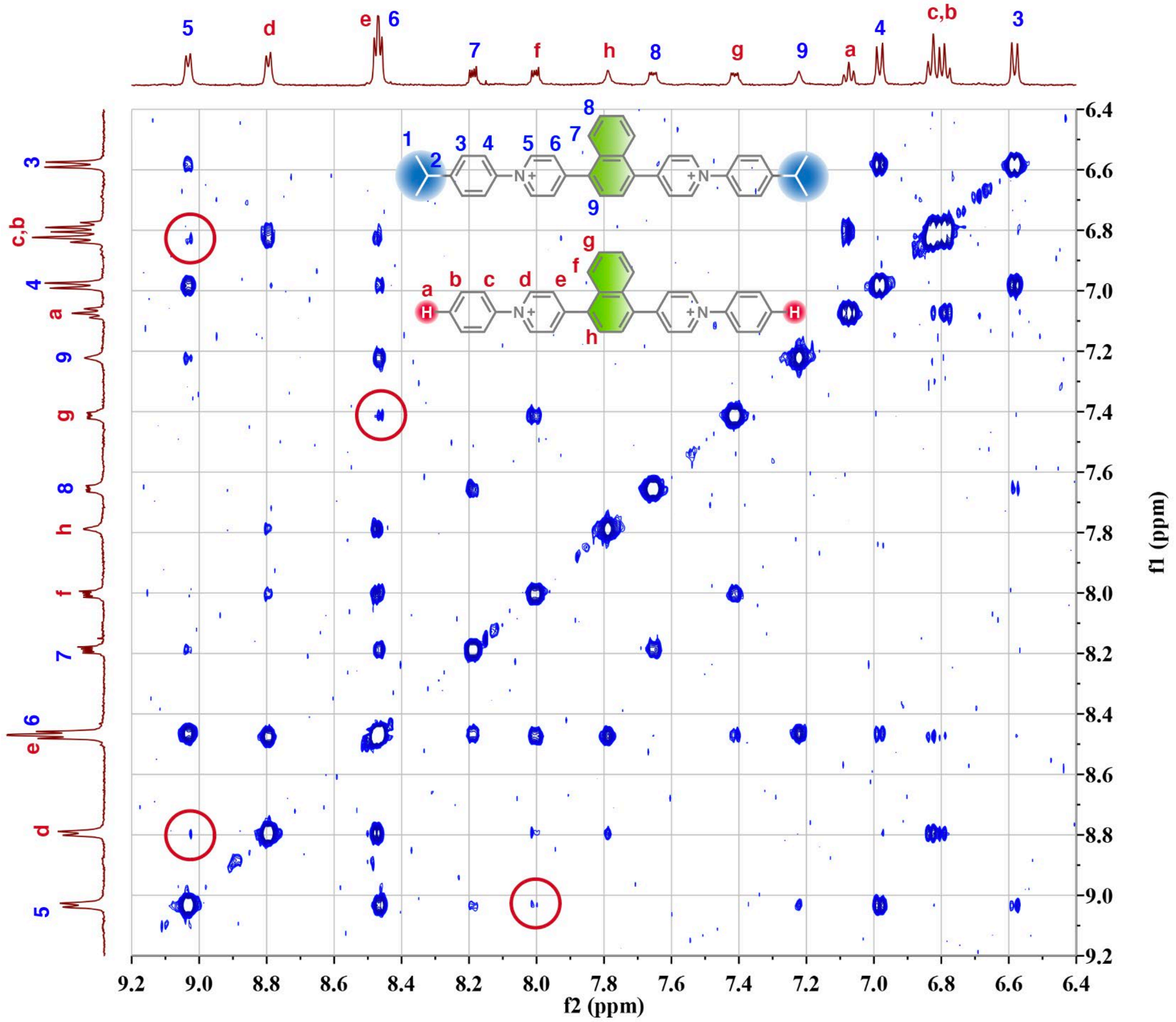

Figure S12. NOESY spectrum of **Np14H• Np14CMe$_2$•CB[8]$_2$** heterodimer in D_2O at 298 K (mixing time: 1000 ms).

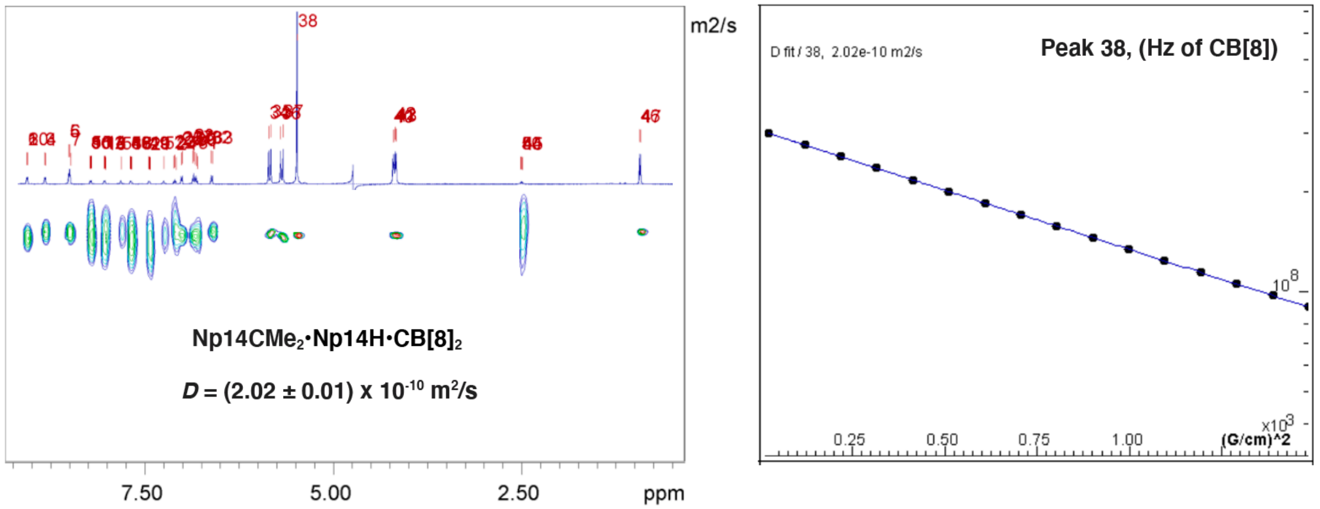

Figure S13. DOSY analysis for **Np14H• Np14CMe$_2$•CB[8]$_2$** heterodimer in D_2O at 298 K, showing one complex with a diffusion coefficient of 2.02 x 10^{-10} m^2/s.

SI-9 Mixture of Ant910H, Np14CMe₂, and CB[8]

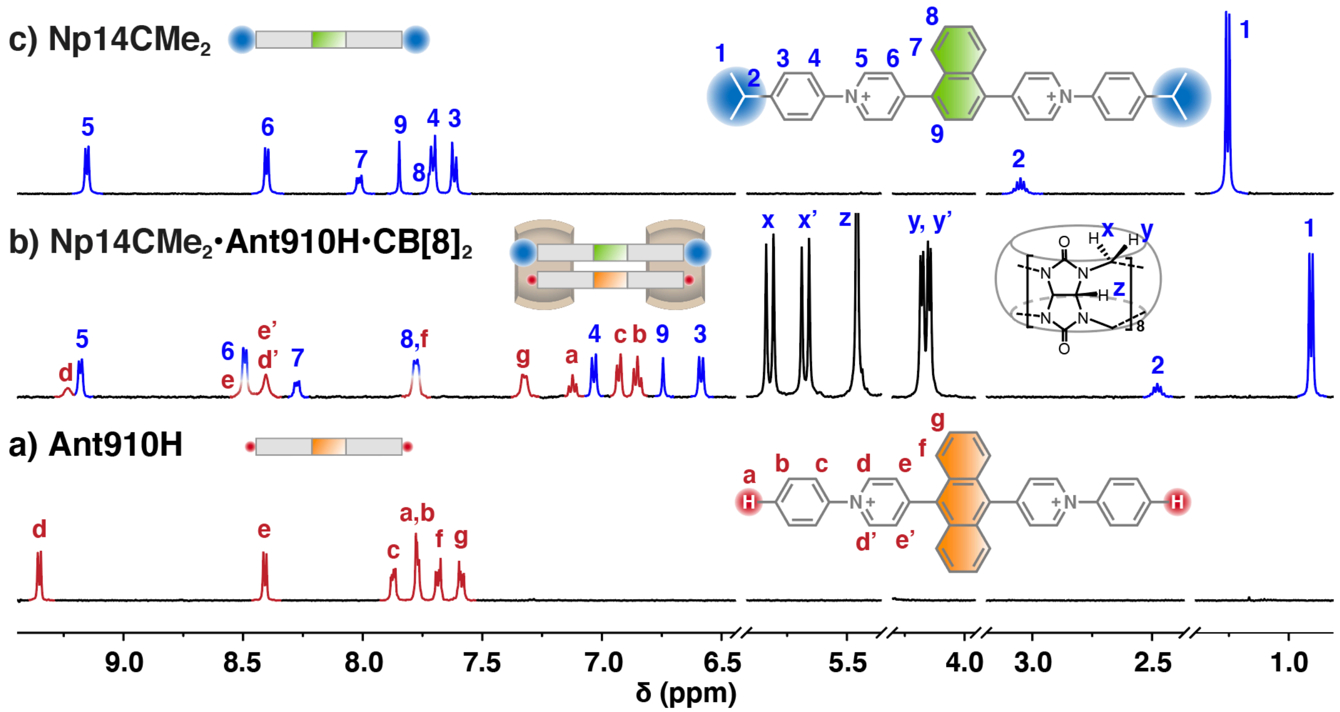

Figure S14. ^{1}H NMR spectra of a) **Ant910H**, b) **Np14CMe₂•Ant910H• CB[8]₂**, c) **Np14CMe₂** in D_2O at 298 K. Protons are assigned using the COSY and NOESY spectra in Figure S16-17.

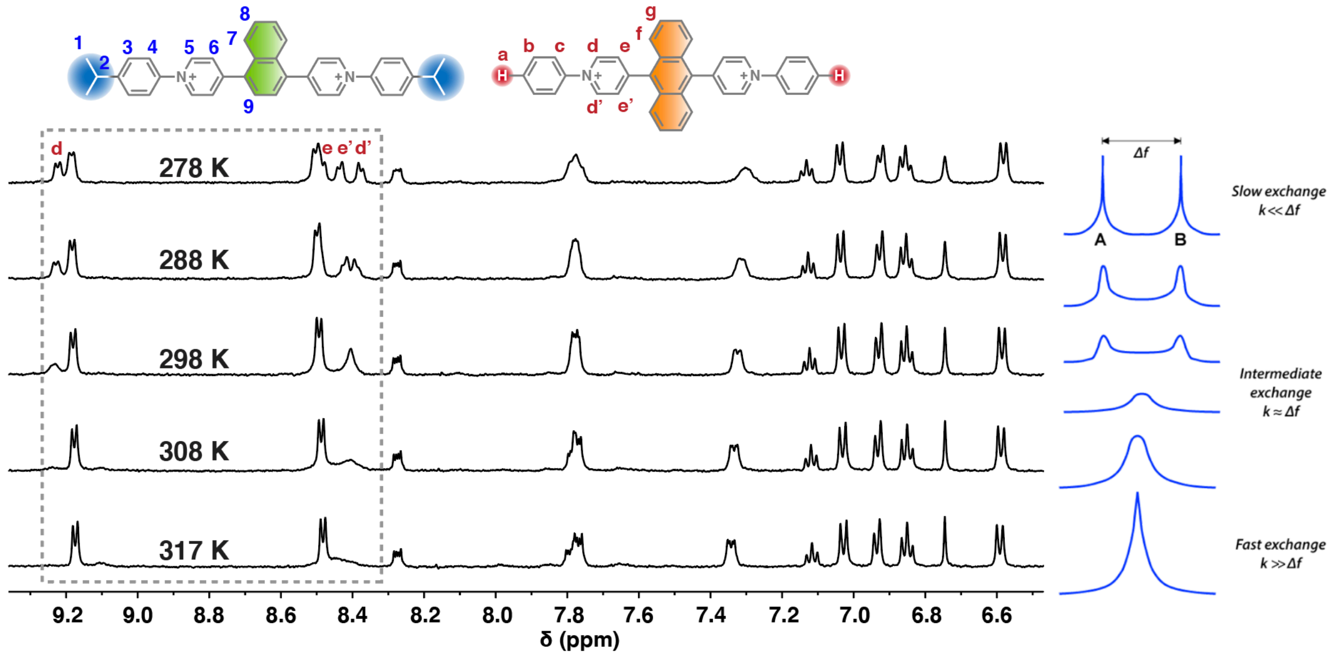

Figure S15. VT-NMR spectra of **Np14CMe₂•Ant910H• CB[8]₂** in D_2O.

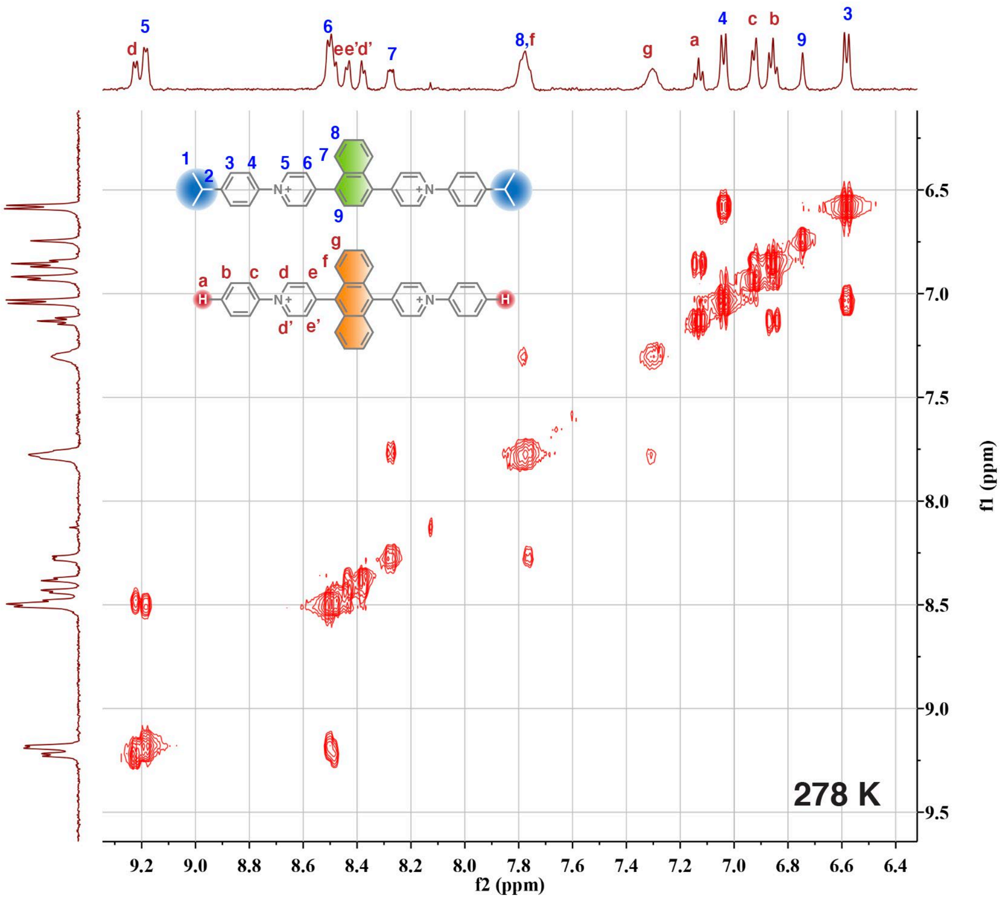

Figure S16. COSY spectrum of **Np14CMe$_2$•Ant910H• CB[8]$_2$** heterodimer in D_2O at 278 K.

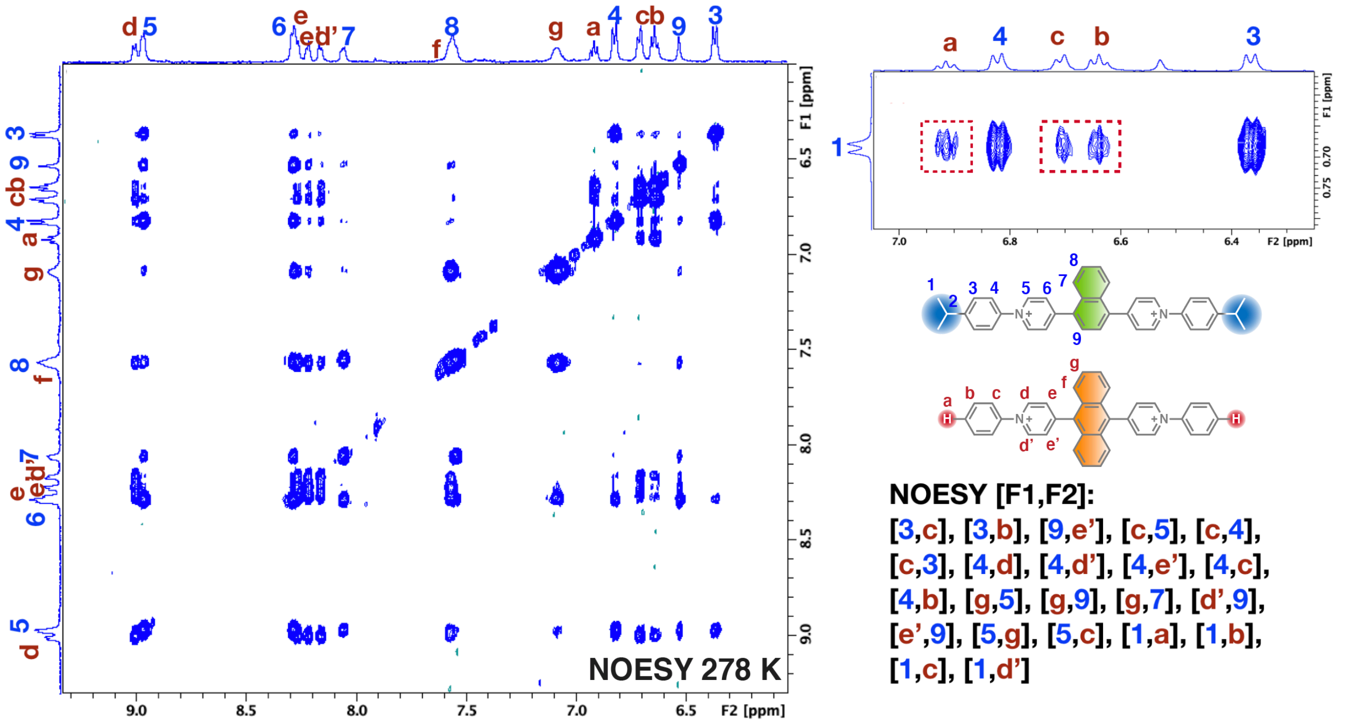

Figure S17. NOESY spectrum of **Np14CMe$_2$•Ant910H• CB[8]$_2$** heterodimer in D_2O at 278 K (mixing time: 1200 ms). Correlation signals are easier to be observed under low temperature.

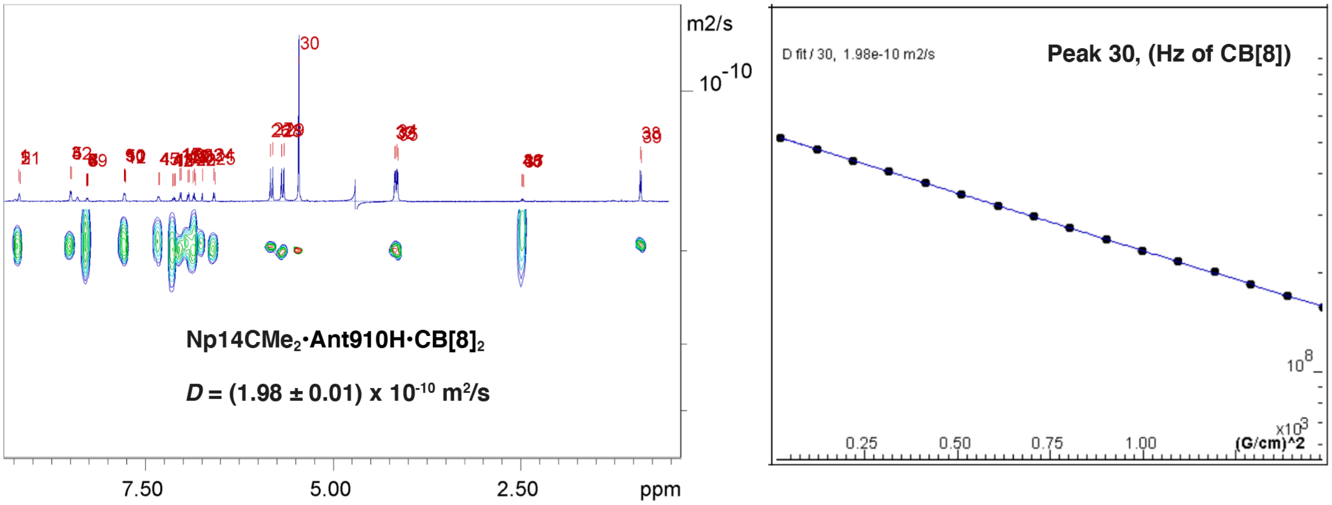

Figure S18. DOSY analysis for **Np14CMe$_2$•Ant910H• CB[8]$_2$** heterodimer in D_2O at 298 K, showing one complex with a diffusion coefficient of 1.98 x 10^{-10} m^2/s.

SI-10 Mixture of Np14H, Ant910CMe₂, and CB[8]

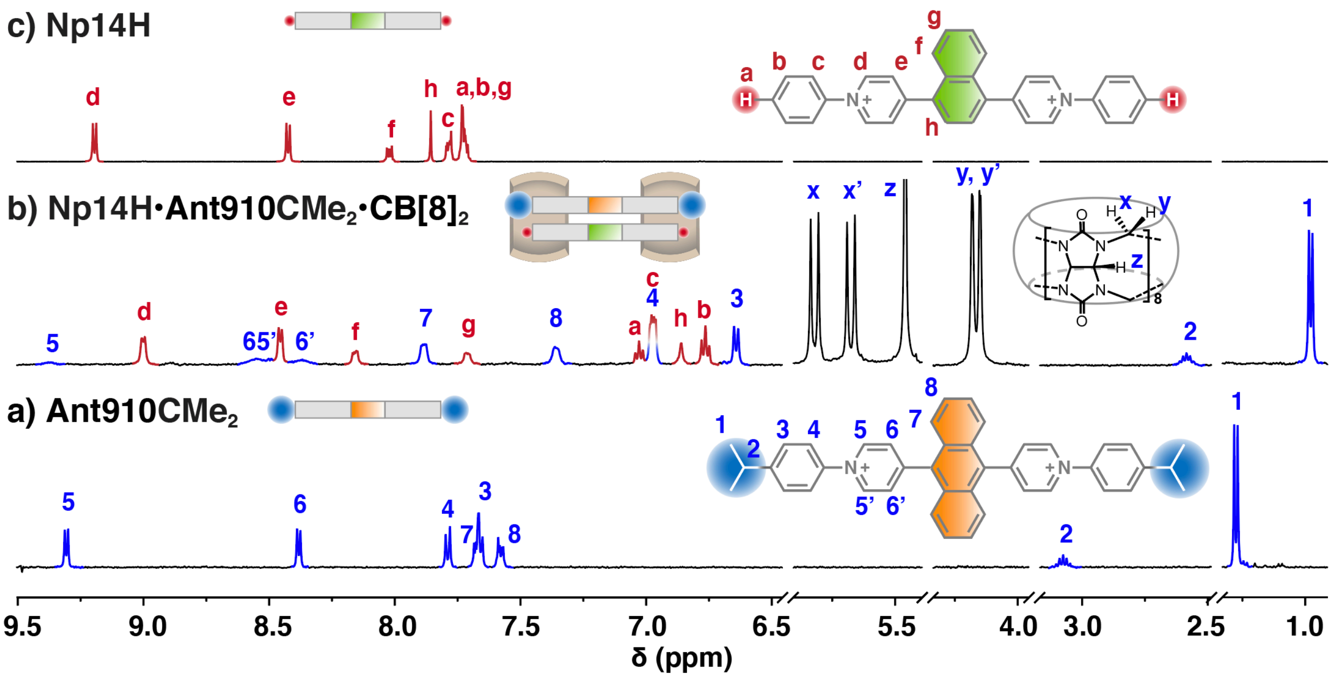

Figure S19. ^{1}H NMR spectra of a) **Np14H**, b) **Np14H• Ant910CMe$_2$•CB[8]$_2$**, c) **Ant910CMe$_2$** in D_2O at 298 K. Protons are assigned using the COSY and NOESY spectra in Figure S21-22.

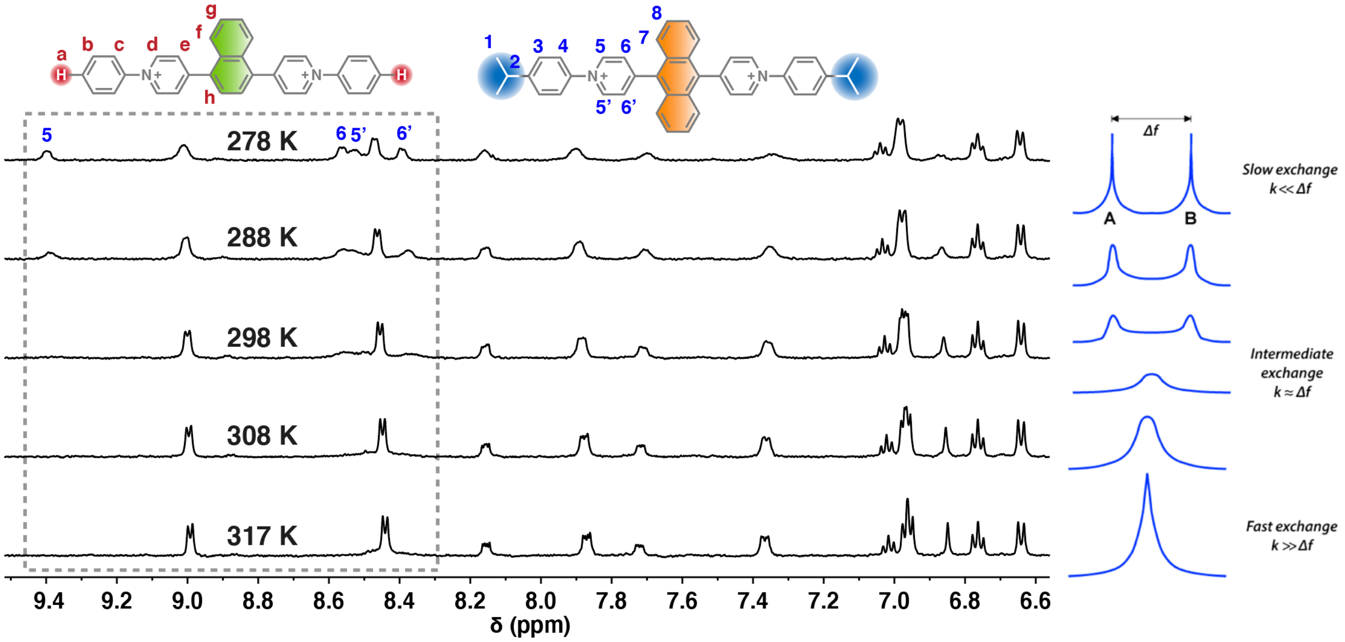

Figure S20. VT-NMR spectra of **Np14H• Ant910CMe$_2$•CB[8]$_2$** in D_2O.

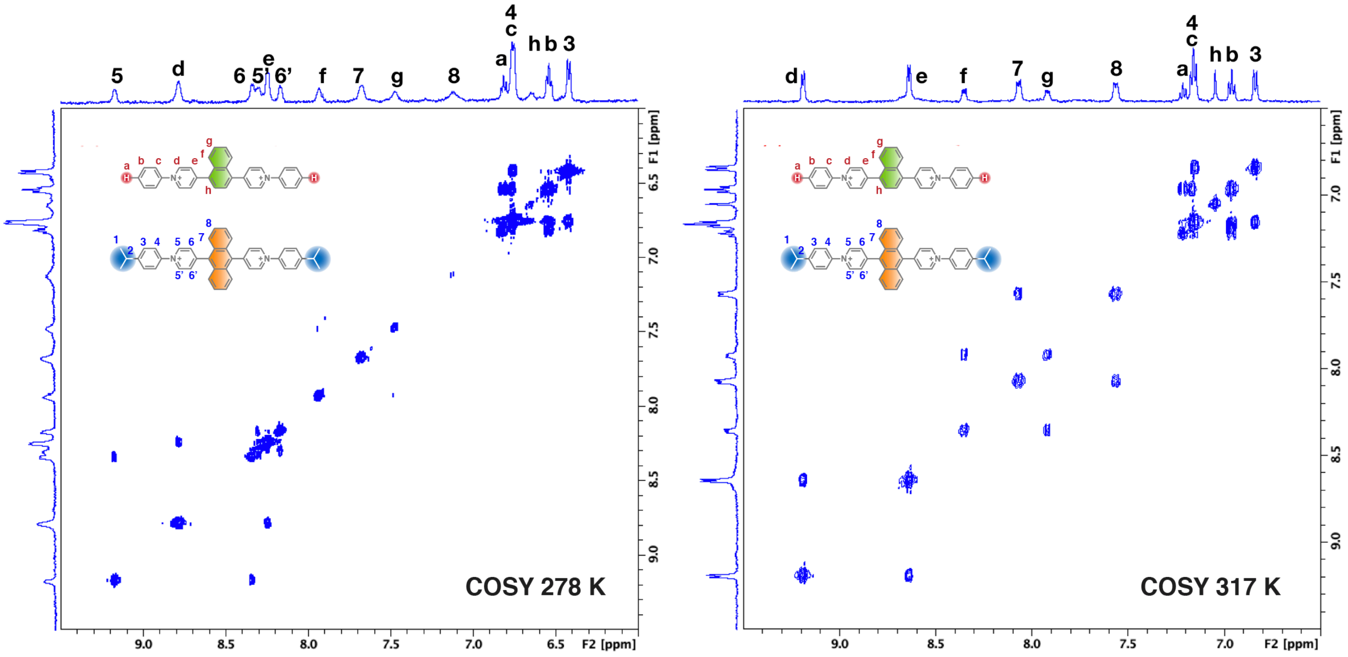

Figure S21. COSY spectra of **Np14H• Ant910CMe$_2$•CB[8]$_2$** heterodimer in D_2O at 278 K and 317 K.

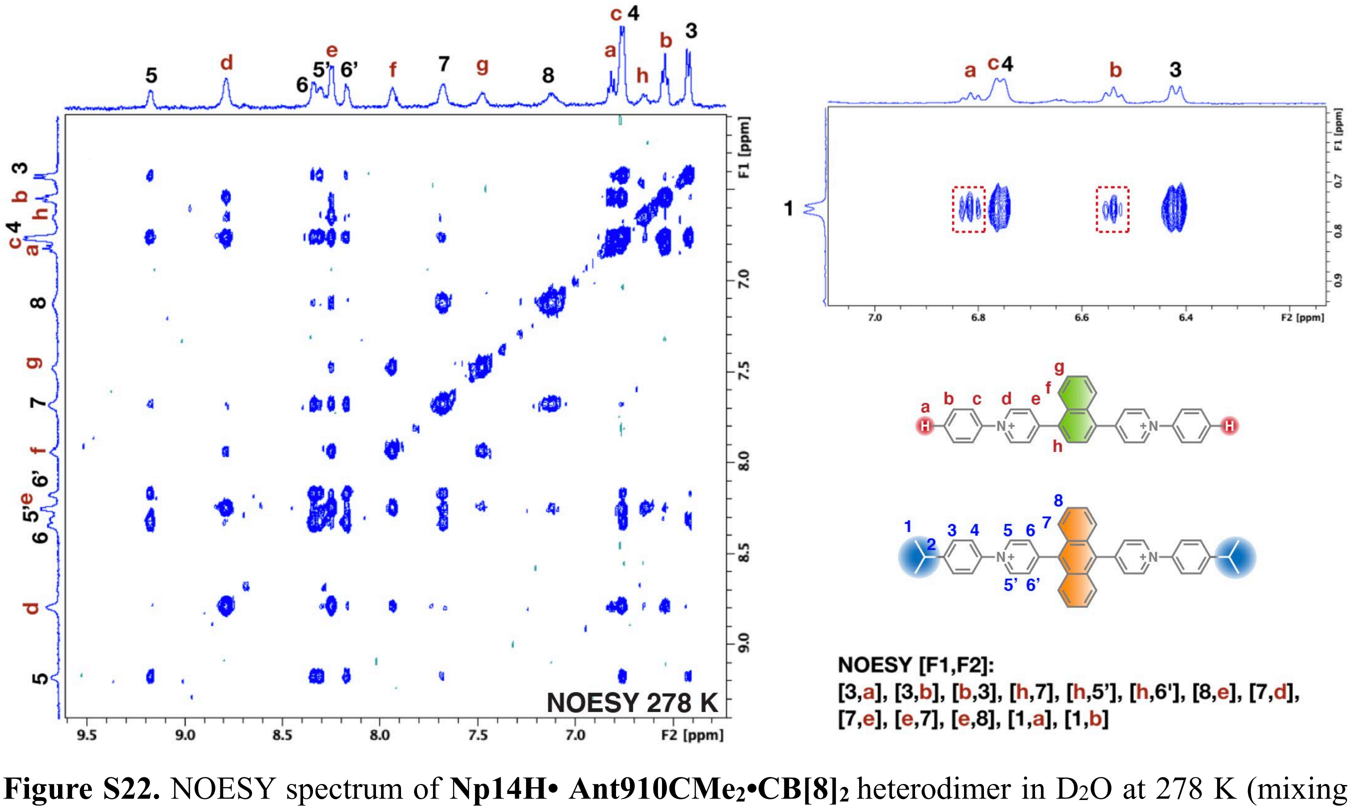

Figure S22. NOESY spectrum of **Np14H• Ant910CMe$_2$•CB[8]$_2$** heterodimer in D_2O at 278 K (mixing time: 1200 ms). Correlation signals are easier to be observed under low temperature.

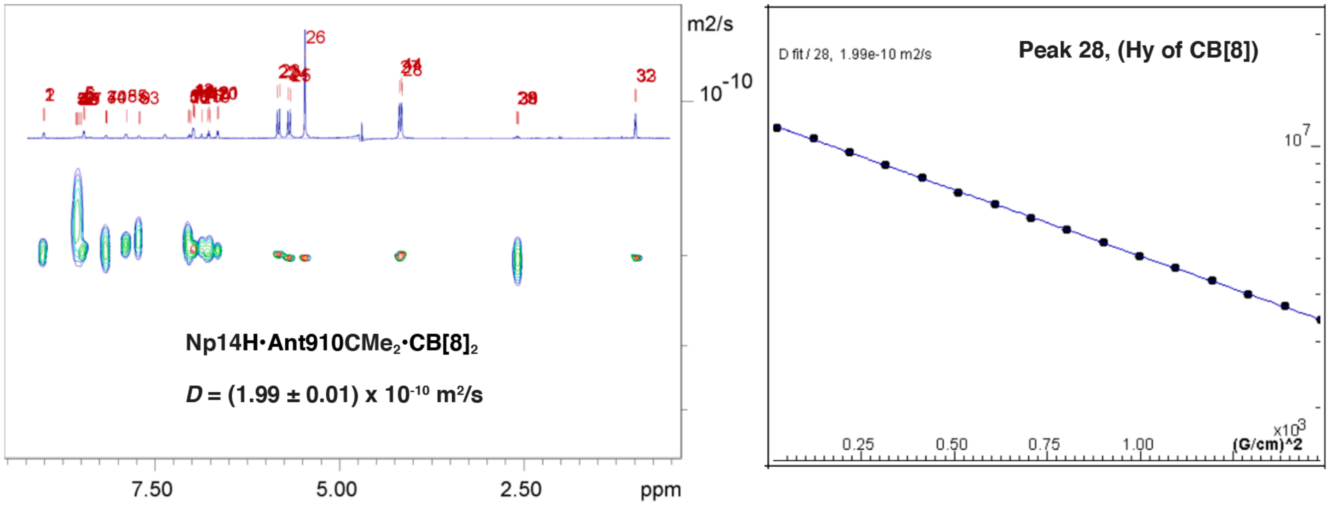

Figure S23. DOSY analysis for **Np14H• Ant910CMe$_2$•CB[8]$_2$** heterodimer in D_2O at 298 K, showing one complex with a diffusion coefficient of 1.99 x 10^{-10} m^2/s.

SI-11 Diffusion coefficient of CB[8]-directed dimers

Table S1. Diffusion coefficient of CB[8]-directed dimers from DOSY in D_2O at 298 K.

Species	*Diffusion Coefficient (10^{-10} · m^2/s)*		
	G_2-CB[8]$_2$[6] or G-G'-CB[8]$_2$	G_2-CB[8]$_3$	G_1-CB[8]$_1$
Ant910Me	2.04 ± 0.01	-	-
Ant15Me	1.98 ± 0.01	-	-
Np27Me	2.00 ± 0.01	-	-
Np14Me	1.98 ± 0.01	-	-
Np15Me	2.00 ± 0.01	-	-
Ph14Me	2.06 ± 0.01	-	-
Ph13Me	2.07 ± 0.01	-	-
Ph135Me	N.D.	1.79 ± 0.01	-
VNMe$_2$	2.01 ± 0.01	-	-
dzpy	-	-	3.04 ± 0.01
CB[8]	-	-	3.11 ± 0.01
Np14H	2.06 ± 0.01	-	-
Np14H-Np14NMe$_2$	2.00 ± 0.01	-	-
Np14H-Np14CMe$_2$	2.02 ± 0.01	-	-
Np14CMe$_2$-Ant910H	1.98 ± 0.01	-	-
Np14H-Ant910CMe$_2$	1.99 ± 0.01	-	-

SI-12 Complexation of Ant910H and Np14CMe$_2$ with CB[7]

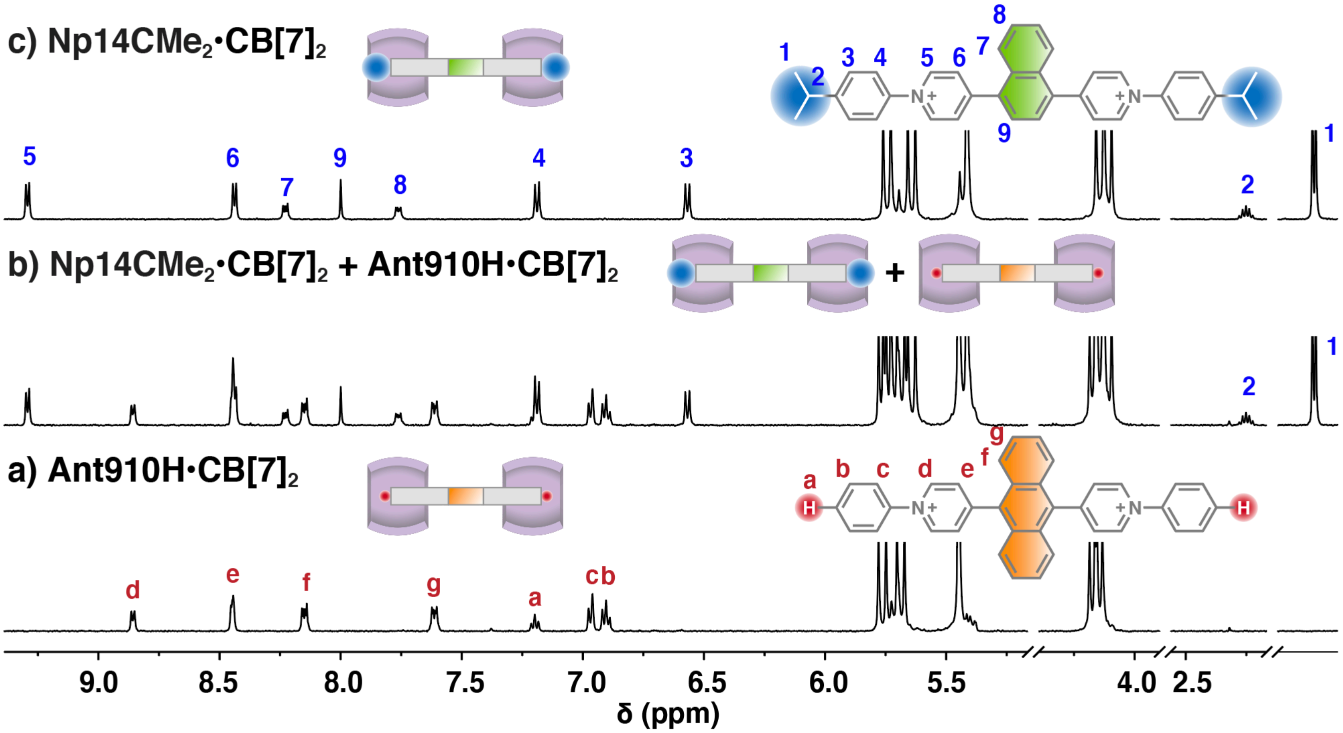

Figure S24. ^{1}H NMR spectra of a) **Np14CMe$_2$•CB[7]$_2$**, c) **Ant910H•CB[7]$_2$**, and b) the equilibrium products of a 50/50 mixture of **Np14CMe$_2$•CB[7]$_2$** and **Ant910H•CB[7]$_2$** in D_2O at 298 K.

SI-13 Excitation spectrum of Ant910H and Np14CMe₂ dimers

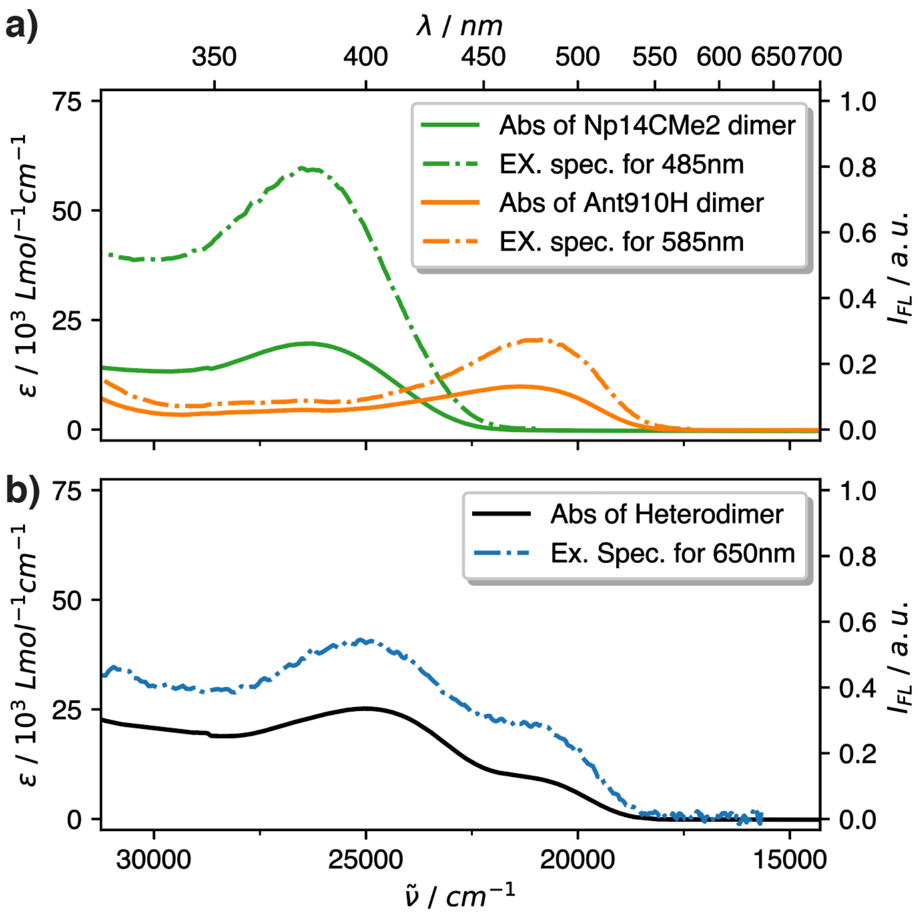

Figure S25. Absorption (solid line) and excitation spectra (dash dotted line) of a) **Np14CMe$_2$** (green) or **Ant910H** (orange) homodimers and b) **Np14CMe$_2$•Ant910H• CB[8]$_2$** heterodimer in D_2O (20 μM). The excitation spectra of **Np14CMe$_2$•Ant910H• CB[8]$_2$** was detected at 650 nm, which corresponds to the emission solely from anthracene moiety without interference from naphthalene. The intense excitation band around 400 nm confirms an energy-transfer contribution from **Np14CMe$_2$** to the emission of **Ant910H**.

Reference

[1] Day, A.; Arnold, A. P.; Blanch, R. J.; Snushall, B. *J. Org. Chem.* **2001**, *66*, 8094–8100.

[2] Bongard, D.; Möller, M.; Rao, S. N.; Corr, D.; Walder, L. *Helv. Chim. Acta* **2005,** *88,* 3200–3209.

[3] Biedermann, F.; Scherman, O. A. *J. Phys. Chem. B* **2012**, *116* (9), 2842–2849.

[4] Zhang, W.; Gan, S.; Vezzoli, A.; Davidson, R. J.; Milan, D. C.; Luzyanin, K. V.; Higgins, S. J.; Nichols, R. J.; Beeby, A.; Low, P. J.; Li, B.; Niu, L. *ACS Nano* **2016**, *10*, 5212–5220.

[5] Wu, G.; Olesińska, M.; Wu Y.; Matak-Vinkovic D.; Scherman, O. A. *J. Am. Chem. Soc.* **2017**, *139*, 3202–3208

[6] Wu, G.; Bae, Y. J.; Olesińska, M.; Antón-García, D.; Szabó, I.; Rosta, E.; Wasielewski, M. R.; Scherman, O. A. *Chem. Sci.* **2020,** *11,* 812–825.

[7] Olesińska, M.; Wu, G.; Gómez-Coca, S.; Antón-García, D.; Szabó, I.; Rosta, E.; Scherman, O. A. *Chem. Sci.* **2019**, *10,* 8806–8811.
